# Leadership education to support person-centred health and social care: A scoping review of empirical literature

**DOI:** 10.1371/journal.pone.0350991

**Published:** 2026-06-08

**Authors:** Qarin Lood, Ewa Carlsson Lalloo, Jana Bergholtz, Emmelie Barenfeld

**Affiliations:** 1 Department of Health and Rehabilitation, Institute of Neuroscience and Physiology, Sahlgrenska Academy, University of Gothenburg, Gothenburg, Sweden; 2 Centre for Person-Centred Care (GPCC), Sahlgrenska Academy, University of Gothenburg, Gothenburg, Sweden; 3 Department of Caring Science, Faculty of Caring Science, Work Life, and Social Welfare, University of Borås, Borås, Sweden; 4 Institute of Health and Care Sciences, Sahlgrenska Academy, University of Gothenburg, Gothenburg, Sweden; Mutah University, JORDAN

## Abstract

The purpose of this scoping review was to map evidence on leadership education to support person-centred care. Specifically, the review aimed to identify the key content of such education, the educational methods used, the reported results, and gaps in the existing literature. The review was conducted using the Joanna Briggs Institute methodology for scoping reviews and reported in accordance with PRISMA-ScR guidelines. Peer-reviewed empirical literature on leadership education for people with leadership roles in health and social were included, without restrictions on publication year or study design. Qualitative, quantitative, and mixed-methods designs were eligible. Educational methods were summarised descriptively, and education content and results were iteratively synthesised and categorised. Methodological quality was not formally assessed, consistent with scoping review methodology. Of 2548 identified records, 22 publications met the eligibility criteria. Leadership education interventions were predominantly conducted in Northern Europe and mainly targeted registered nurses in leadership roles. Educational content commonly addressed leadership theories and styles, person-centredness, and facilitation skills. Educational methods were largely based on longitudinal, work-based learning approaches, and reported results primarily reflected perceived improvements in leadership practices, workplace culture, and care practice outcomes. However, outcomes were largely self-reported, context-specific, and seldom assessed using standardised or comparative measures. Interpretation of the findings is limited by the small number of publications, the predominance of qualitative and non-comparative designs, and the narrow geographical and professional focus of the evidence. These limitations restrict conclusions regarding effectiveness, generalisability, scalability, and sustained system-level impact. Despite these limitations, the findings suggest that leadership education may support leadership practices and care improvement. The review identifies substantial gaps in the global evidence base, highlighting the need for more diverse, theory-informed, and rigorously evaluated leadership education that engages multiple professional groups, incorporates patient and relative perspectives, and examines implementation and transferability across varied health and social care contexts.

## Introduction

Person-centred care is widely recognised as a core principle of high-quality healthcare, with growing support for its systematic implementation worldwide. The World Health Organization (WHO) [1] identifies person-centredness as a core competency for healthcare professionals, a key element of primary care, and a key enabler of Universal Health Coverage [1]. Although person-centred care is often described at the level of clinical encounters, its implementation requires transformation at organisational and system levels, extending beyond individual professional practice [2].

In contrast to traditional health and social care models that prioritise disease and causes of suffering [3], person-centred care represents a philosophical and cultural emphasis on partnership, values, and relational processes [2]. While related approaches such as patient-centred care focus primarily on functional goals, person-centred care is oriented towards meaningful goals grounded in people’s lives [4]. Importantly, person-centred care extends beyond care encounters to include leadership and organisational practices [2], distinguishing it from other “centred” models that focus mainly on the patient-professional interaction [5]. McCormack and McCance [6] define person-centred care as practice grounded in respect for persons, self-determination, and mutual understanding, built upon and sustained through therapeutic relationships among leaders, direct care staff, patients, and relatives. Similarly, Ekman et al. [7] conceptualise person-centred care as an ethical approach that recognises patients as active partners with resources and capabilities [7]. Together, these perspectives underscore that person-centred care is not merely a clinical method but an ethical orientation that must be supported structurally and culturally within organisations.

Both management and leadership are critical for achieving organisational goals such as implementing person-centred care [2,8]. However, their functions differ. While management focuses on stability, coordination, and control, leadership facilitates change, sense-making, and collective direction [9,10]. Because person-centred care challenges established professional hierarchies and routines, its implementation places particular demands on leadership, requiring leaders to actively embody and support person-centred values in everyday practice [2,8]. This review therefore focuses on leadership education targeting people in formal leadership roles, positioned to influence organisational priorities, cultures, and learning environments.

Leading towards person-centred care often requires rethinking traditional leadership approaches. Person-centred care challenges conventional command-and-control models and calls for leadership that is relational and inclusive [11]. Leaders must navigate tensions between regulatory requirements, resource constraints, and the ethical imperative to collaborate meaningfully with patients, relatives, and direct care staff [12]. Leadership in health and social care is further complicated by its distributed nature, with influence and decision-making shared across professional and organisational boundaries [13]. Accordingly, leadership for person-centred care is increasingly conceptualised as both an individual and collective practice, aligning with theories of shared, relational, and practice-based leadership [13–15]. This complexity may contribute to limited clarity regarding what is required to lead effectively towards person-centred care and how leaders can be supported to do so [2,8,16]. Backman et al. [8] describe person-centred leadership as integrating “being” and “doing”, combining values, self-awareness, and relational competence with concrete leadership actions. Similarly, Deuling et al. [17] conceptualise leadership as encompassing both formal roles and individual qualities and skills. Within a person-centred care context, leadership involves promoting a person-centred vision and culture, serving as a role model, providing commitment and support, engaging patients, and creating forums for person-centred care. These components span interactions at multiple levels, including self, colleagues, patients, direct care staff, and the organisation. Despite these conceptual advances, there remains limited empirical evidence on how such leadership capabilities can be developed through education and training [17].

Leadership education therefore represents a critical, yet underdeveloped, means for advancing person-centred care. Leaders in complex health and social care systems require educational support that develops not only technical and managerial skills but also interpersonal, ethical, and reflexive competencies [16,18]. Effective leadership development is known to combine formal education with workplace-based and informal learning to support transfer into practice [19]. Existing literature on leadership education aimed at supporting person-centred care has primarily targeted health and social care staff rather than persons in formal leadership roles [20], leaving limited clarity regarding which content and educational methods are most effective for supporting the transition towards person-centred care [8,17].

Finally, leadership education for person-centred care must be understood within a global context. Health and social care systems differ substantially in governance, professional roles, resources, and cultural expectations, all of which shape leadership practices and educational needs. These variations raise important questions about the transferability of leadership education developed in specific contexts and highlight the need to map existing evidence across diverse settings. Given the emerging and heterogeneous nature of this field, a scoping review is an appropriate approach. As described by the Peters et al. [21] scoping reviews aim to map the extent, range, and nature of evidence rather than to test hypotheses or evaluate intervention effectiveness [21]. Accordingly, this review aimed to map existing evidence on leadership education designed to support person-centred care, identify knowledge gaps, and inform future research and educational development. The review addressed the following research questions:

- What is described as key content of leadership education to support person-centred care?
- Which educational methods have been used?
- Which results have been reported?
- What knowledge gaps exist in the existing literature?

## Materials and methods

### Study design and registration

A scoping review was conducted in accordance with the Joanna Briggs Institute (JBI) methodology [21] and reported following the Preferred Reporting Items for Systematic Reviews and Meta-Analyses Extension for Scoping Reviews (PRISMA-SCR) [22]. A review protocol was registered prospectively in the Open Science Framework (OSF https://doi.org/10.17605/OSF.IO/NYHXZ). In line with JBI guidance [21], no formal appraisal of methodological quality or risk of bias was undertaken, as the aim of the review was to map the extent and nature of the evidence rather than to assess effectiveness.

A reference group comprising patient and carer representatives, representatives from Swedish regional and municipal authorities, and the Swedish Association of Health Professionals was involved throughout the review process. One member of the reference group (JB) is also a co-author. The group contributed to refining the review scope, research questions, eligibility criteria, and presentation, interpretation and dissemination of findings.

### Information sources

Systematic searches were conducted in CINAHL (EBSCO), ERIC (EBSCO), PubMed, and Scopus by an information specialist at the Gothenburg University library. Searches were initially performed on 6 May 2024 and updated on 11 February 2025.

### Eligibility criteria

Eligibility criteria were defined using the Population-Concept-Context framework [21]:

- *Population:* People with formal leadership responsibilities (operative and/or strategic level) in health and social care organisations
- *Concept:* Education aiming to prepare and support leaders in guiding the transition towards person-centred care
- *Context:* Health and social care settings worldwide

Publications were included if they:

- Reported empirical data (qualitative, quantitative, or mixed methods)
- Focused primarily on people in formal leadership roles
- Described leadership education explicitly linked to the implementation of person-centred care
- Were indexed in the selected databases, including indexed grey literature, and
- Were published in English, Finnish, Swedish, Norwegian, Danish or German

Publications were excluded if they were literature reviews or conference abstracts. Language restrictions reflected the languages mastered by the reviewer team to ensure accurate interpretation without external translation.

### Search strategy

The search strategy was developed collaboratively by the research team, reference group, and information specialist, guided by the Population-Concept-Context framework [21]. Controlled vocabulary (e.g., Medical Subject Headings (MeSH terms)) and free-text terms related to leadership, education, and person-centred care were combined using Boolean operators. Full search strategies are presented in Table 1.

**Table 1:** Detailed search strategies used in the review.

#	Searches	Results
**CINAHL (EBSCO)**		
#1	(MH leadership) OR (TI lead* OR AB lead*) OR (MH “Nurse Executives+”) OR (MH “Nursing Leaders+”) OR (MH “Clinical Governance+”) OR (MH “Physician Executives”) OR (MH “Executive Function”) OR (MH “Nurse Managers+”) OR (TI governance OR AB governance) OR (TI manager* OR AB manager*) OR (TI executive* OR AB executive*) OR (TI director* OR AB director*) OR (MH “Decision Making, Organizational”) OR (TI coach OR AB coach) OR (MH mentorship) OR (TI mentor* OR AB mentor*)	124 472
#2	(MH Education+) OR (TI educat* OR AB educat*) OR (TI training OR AB training) OR (MH “staff development+”) OR (TI “staff development*” OR AB “staff development*”) OR (MH “Seminars and Workshops+”) OR (TI workshop* OR AB workshop*) OR (MH lecture) OR (TI lecture* OR AB lecture*) OR (TI course* OR AB course*) OR (MH learning+) OR (TI learning OR AB learning) OR (MH “Quality Improvement+”) OR (TI “quality improvement*” OR AB “quality improvement*”) OR (TI change OR AB change) OR (TI implement* OR AB implement*)	537 748
#3	(TI “Person centered” OR AB “Person centered”) OR (TI “person centred” OR AB “person centred”) OR (TI person-centered OR AB person-centered) OR (TI person-centred OR AB person-centred) OR (TI “person centeredness” OR AB “person centeredness”) OR (TI personcenteredness OR AB personcenteredness) OR (TI “person centredness” OR AB “person centredness”) OR (TI person-centeredness OR AB person-centeredness) OR (TI person-centredness OR AB person-centredness)	2 285
#4	#1 AND #2 AND #3	195
#5	Limit English^1^	194
**ERIC (EBSCO)**		
#1	(DE leadership) OR (TI lead* OR AB lead*) OR (DE Leaders) OR (DE Leadership) OR (DE Governance) OR (TI governance OR AB governance) OR (TI manager* OR AB manager*) OR (TI executive* OR AB executive*) OR (TI director* OR AB director*) OR (TI coach OR AB coach) OR (DE mentors) OR (TI mentor* OR AB mentor*)	243 684
#2	(DE Education) OR (TI educat* OR AB educat*) OR (DE training) OR (TI training OR AB training) OR (DE “staff development”) OR (TI “staff development*” OR AB “staff development*”) OR (DE Workshops) OR (TI workshop* OR AB workshop*) OR (TI lecture* OR AB lecture*) OR (TI course* OR AB course*) OR (DE learning) OR (TI learning OR AB learning) OR (TI “quality improvement*” OR AB “quality improvement*”) OR (TI change OR AB change) OR (TI implement* OR AB implement*)	1 260 471
#3	((TI “Person centered” OR AB “Person centered”) OR (TI “person centred” OR AB “person centred”) OR (TI person-centered OR AB person-centered) OR (TI person-centred OR AB person-centred) OR (TI “person centeredness” OR AB “person centeredness”) OR (TI personcenteredness OR AB personcenteredness) OR (TI “person centredness” OR AB “person centredness”) OR (TI person-centeredness OR AB person-centeredness) OR (TI person-centredness OR AB person-centredness))	1 180
#4	#1 AND #2 AND #3	101
#5	Limit English^1^	94
**Pubmed**		
#1	leadership[mesh] OR lead*[tiab] OR governance[tiab] OR manager*[tiab] OR executive*[tiab] OR director*[tiab] OR Decision Making, Organizational[mesh] OR coach[tiab] OR mentors[mesh] OR mentor*[tiab]	2 268 688
#2	Education[mesh] OR educat*[tiab] OR Inservice training[mesh] OR training[tiab] OR staff development[mesh] OR staff development[tiab] program*[tiab] OR workshop*[tiab] OR lecture*[tiab] OR course*[tiab] OR learning[mesh] OR learning[tiab] OR Quality Improvement[mesh] OR quality improvement*[tiab] OR change[tiab] OR implement*[tiab]	3 741 718
#3	Person centered[tiab] OR person centred[tiab] OR person-centered[tiab] OR person-centred[tiab] OR person centeredness[tiab] OR personcenteredness[tiab] OR person centredness[tiab] OR person-centeredness[tiab] OR person-centredness[tiab]	11 366
#4	#1 AND #2 AND #3	826
#5	Limit English^1^	818
**Scopus**		
#1	TITLE-ABS-KEY(leader* OR governance OR manager* OR executive* OR director* OR coach OR mentor*)	1 535 757
#2	TITLE-ABS-KEY(educat* OR “Inservice training” OR training OR “staff development” OR workshop* OR lecture* OR course* OR learning OR “quality improvement*” OR change OR implement*)	18 188 111
#3	TITLE-ABS-KEY(“Person centered” OR “person centred” OR “person centeredness” OR personcenteredness OR “person centredness” OR person-centeredness OR person-centredness)	17 537
#4	#1 AND #2 AND #3	1 032
#5	Limit English^1^	1 025

^1^English was used as limitation in the search strategy since there were no publications in the other languages mastered by the reviewer team (Danish, Finnish, German, Norwegian or Swedish).

Although “patient-centred care” is an established MeSH term, it was not included, as the review focused explicitly on person-centred care as a broader ethical and relational care approach encompassing patients, relatives, direct care staff, and leaders across the continuum of care and organisations, rather than primarily clinical encounters [11,23]. To minimise the risk of missing relevant studies, reference lists of included publications were manually screened. No restrictions were applied regarding year of publication.

### Screening and selection of publications

References were managed in Endnote, and duplicates were removed prior to screening. Title and abstract screening was conducted using Rayyan software. Following Levac et al. [24], the eligibility criteria were pilot-tested on a random sample of 25 titles and abstracts by three reviewers (ECL, JB, QL). Subsequently, two reviewers (ECL and JB or JB and QL) independently screened titles and abstracts, with discrepancies resolved through consultation with a third reviewer (EB). Publications without abstracts were forwarded directly to full-text screening. Full-text screening was conducted independently by ECL and JB, with disagreements resolved through discussion and involvement of QL.

### Data extraction

Data were extracted independently by four reviewers (QL, JB, EB, ECL) using a structured Microsoft Excel form developed for this review. Extracted data included: title, authors, year of publication, country where the empirical work was conducted, study design, aims(s), context, descriptions of leadership and person-centred care, participant characteristics, key content, educational methods, summary of results, involvement of patients, relatives, or other interest holders, and knowledge gaps, including suggestions for future research and reported methodological limitations.

Although the initial plan focused on patient and public involvement, it became evident that involvement of direct care staff and other interest holders was also relevant. Data on all involvement activities were therefore extracted under the broader category of interest holder involvement, with interest holders defined according to Akl et al. [25], as persons or groups with legitimate concerns regarding health issues, including staff, patients, relatives, members of the public, and policymakers.

### Data analysis

A descriptive qualitative analysis was conducted in line with scoping review methodology [21]. Following data extraction, the first author organised data according to the research questions (key content, educational methods, reported results, and knowledge gaps). Text segments were coded based on manifest content and iteratively grouped into descriptive categories where appropriate to provide an overview of the scope and characteristics and scope of leadership education for person-centred care. To enhance analytical rigour and transparency, preliminary categories were discussed among all authors and with the reference group and refined through iterative dialogue until consensus was reached. As the purpose of the review was mapping rather than interpretative synthesis, no theory-building or latent interpretation was undertaken, and inter-rater reliability statistics were not calculated. Credibility was supported through reviewer triangulation and collaborative validation of categories. Data on publication and participant characteristics, interest holder involvement, and knowledge gaps were summarised narratively and presented in text and tables to provide contextual detail and support transparency.

### Ethics statement

The authors have nothing to report.

## Results

**Fig 1:**
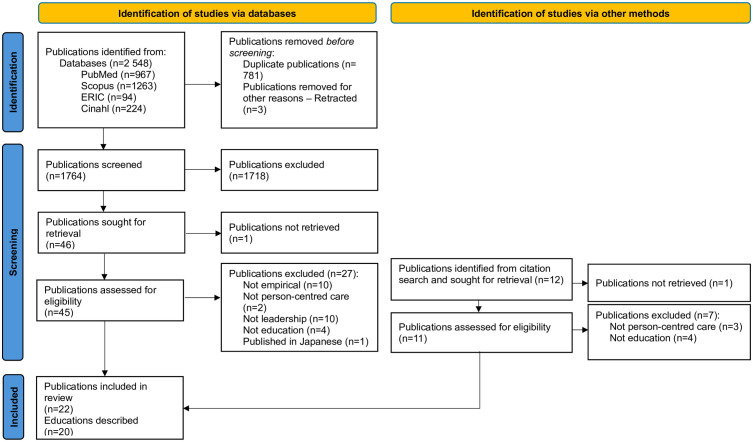
PRISMA flow chart. PRISMA flow chart of the study selection process for the scoping review, reported in accordance with PRISMA-ScR guidelines [22].

The database search yielded 2548 records (Fig. 1). After removal of duplicates and retracted publications, 1764 records remained for title and abstract screening. Following this initial screening, one publication was removed as it could not be retrieved, and 45 publications remained for full-text screening. This process resulted in 18 publications that met the eligibility criteria. Manually searching of reference lists identified an additional four publications, resulting in a final sample of 22 publications describing 20 distinct educational interventions.

The included publications were published between 2001 and 2025 and were conducted in various health and social care settings primarily in Northern Europe (n = 19), with additional publications from Australia (n = 2) and the United States of America (n = 1). Study designs were heterogeneous: ten publications used action research designs, four employed qualitative designs, three used experimental designs, and five applied other designs (case study, cross-sectional, or mixed methods). Interest holder involvement (staff, patients, relatives, and other interest holders), was reported in relation to the development and/or evaluation of the educational interventions. An overview of publication characteristics is provided in Table 2.

**Table 2:** Overview of characteristics of included publications, in chronological order.

Author(s), year, ref.	Setting	Research aim(s)	Research methods	Interest holder involvement
Wright and McCormack, 2001 [26]	Hospital in the United Kingdom	To describe the development of a person-centred approach to working with older people.	Practice development derived from action learning methodology	The study had a collaborative nature, including a steering group with key interest holders (members of multidisciplinary and hospital manager). During the study, an action plan was established with nurses and a multidisciplinary team.
Mansell et al., 2008 [27]	Community intellectual disability residential homes in England	To study engagement, care practices and a range of staff and organisational characteristics.	Controlled trial	Not mentioned.
Boomer and McCormack, 2010 [28]	Wards and clinics in the United Kingdom	To evaluate a 3-year practice development programme for clinical nurse leaders.	Programme evaluation methodology	The project included patient and staff nurse panels and a peer advisory group.
Cardiff 2012 [29]	Hospital in the Netherlands	To describe and evaluate a critical and creative reflective inquiry structure and processes.	Participatory action research	The study’s participatory nature involved communicating research results to the team, planning action cycles, and leaders facilitating storytelling sessions with staff.
Manley et al., 2014 [30]	Various health services in the United Kingdom	To review specialist practice and how this informed the development of a trust wide, shared purpose framework relevant to all staff.	Practice development (action research)	The study employed workplace inquiry and participatory research principles. Patients, carers, and service users were included in a feedback process.
Jeon et al., 2015 [31]	Community aged care (residential and home care) in Australia	To evaluate the effectiveness of a leadership and management programme in aged care.	Double-blind cluster randomised controlled trial	Views of aged care key interest holders were documented in a video (registered nurses, managers, education consultant, executive officers).
Brooker et al., 2016 [32]	Care homes in the United Kingdom	To report on the acceptability and effectiveness of the FITS (Focussed Intervention Training and Support) into practice programme.	Mixed methods case study	The project included an external steering group including patient/carer representatives (one of them as co-author), senior clinicians, care home management staff, and inspection and regulation representation.
Eide et al., 2016 [33]	Community healthcare (residential and home care) in Norway	To develop and investigate the feasibility of a 6-week web-based, ethical leadership educational programme and learn from participants’ experience.	Focus groups	Participants were encouraged to reflect upon project ideas before signing up, to involve staff members and to choose a project that both leader and staff together would find interesting.
Manley and Titchen, 2017 [34]	Various healthcare settings in the United Kingdom	To help new and emerging consultants to become more effective in their role through a programme of support to develop their practice.	Collaborative emancipatory action research	The focus of the study was to enable practitioners to become practitioner-researchers.
Bradd et al., 2018 [35]	Various hospital and healthcare facilities in Australia	To examine whether practice development combined with transformational leadership approaches was effective in improving allied health professionals’ ability to lead and manage change intended to improve culture, quality and safety, ways of working and/or person-centred care.	Mixed methods randomised controlled trial	Some of the practice development projects chosen by participants included partnering with patients to improve services.
Cardiff et al., 2018 [36]	Hospital in the Netherlands	To describe how person-centred leadership manifest in clinical nursing.	Participatory action research	Health professionals were involved through collaborative inquiry and as co-researchers. Patients were involved in the orientation phase of the study.
Lynch et al., 2018 [37]	Community residential aged care in Ireland	To implement and evaluate the effect of using the person-centred situational leadership framework to develop person-centred care within nursing homes.	Action research, partly participatory	Older persons and staff were partly involved in developing a framework for person-centred situational leadership through narratives and focus groups.
Dellenborg et al., 2019 [38]	Hospital in Sweden	To explore what may be required for person-centred care programmes to be successful.	Ethnographic method	Not mentioned but highlights the collective nature of learning processes and change.
Hardiman and Dewing, 2019 [39]	Hospital in Ireland	To examine facilitation in workplace learning where nurses are focused on creating person-centred cultures; to provide a framework for novice and proficient facilitators/practitioners to learn in and from their own workplaces and practices; and to provide the conditions where practitioners can gain an understanding of the culture and context within their own workplace.	Participatory action research	The study involved participatory process as part of the study design and a baseline culture assessment was discussed with interest holders (the hospital-wide nursing governance team).
Stanhope et al., 2019 [40]	Community mental health clinics in the United States of America	To explore variation in organisational readiness for change and leadership behaviour across seven organisations during a 12-month training intervention in person-centred care planning.	Longitudinal mixed methods	Not mentioned.
McCormack et al. 2021 [41]	Community healthcare in the United Kingdom	To engage in a participatory evaluation of the experience of the nine-month development journey of the 2019 Queen’s nurse development programme participants.	Collaborative critical creative inquiry methodology	The education was inductively co-created, focusing on inquiring with persons rather than on persons.
McCormack et al., 2022 [42]	Hospital and residential units in Ireland	To develop multi-professional facilitators to lead person-centred culture change within their own services and to provide a means for services to embed person-centred ways of working as the norm for how they do their business.	Emancipatory practice development	The programme was co-designed and delivered by two facilitators working in quality improvement and two researchers; all co-authors of the study.
Cable et al., 2024 [43]	Community healthcare services in the United Kingdom	To illuminate the participants’ experiences of engaging in transformative learning and development and identify the technical and transformative outcomes arising.	Collaborative critical creative inquiry methodology	The education was inductively co-created, focusing on inquiring with persons rather than on persons.
Doody et al. 2024 [44]	Intellectual disability services in Ireland	To design, deliver, and evaluate a leadership programme for nurse and social care managers in Ireland.	Cross-sectional	A healthcare professional [co-author) was involved throughout the study, as well as in the design, delivery and evaluation process of the programme.
Lood et al., 2024 [45]	Various healthcare and social care organisations in Sweden	To explore programme management members’ experiences from the development and realisation of a leadership programme.	Focus groups	The programme was co-developed with healthcare professional representatives.
Årestedt et al. 2024 [46]	Kidney care sites (hospital) in Sweden	To evaluate two implementation strategies: the dissemination of a clinical tool, and additional training and support of internal facilitators for person-centred patient participation in kidney care.	Process evaluation of a quasi-experimental study	Not mentioned.
Hurtig et al. 2025 [47]	Kidney hospital care in Sweden	To illustrate what patient participation signified to patients and staff in kidney care, and whether an agreed or disagreed conceptualisation occurred over time, evaluating the influence of two study-specific interventions to facilitate more person-centred participation.	Convergent mixed methods with interviews and structured reviews of patient records	Not mentioned.

### Overall descriptions of the education interventions

The 20 identified leadership education interventions varied in key content, format, duration, educational methods, theoretical underpinnings, and target groups. Most conceptualised leadership education as a longitudinal, work-based learning process embedded in participants’ organisational contexts rather than short, classroom-based training. Theoretical foundations clustered around leadership and person-centredness. Three main target groups were identified: managers (n = 6), people in non-managerial leadership roles (n = 8), and mixed groups including both managers and other leaders (n = 6). Education participants were predominantly registered nurses holding formal non-managerial leadership roles, and women were overrepresented in publications reporting participant sex. Most interventions (n = 14) extended over six months or longer, particularly those aimed at broader leadership development and cultural change, whereas shorter durations were more common among interventions targeting managers or mixed leadership groups. An overview of key content, educational methods, and reported results is provided in Table 3.

**Table 3:** Brief descriptions of key content, educational methods, and reported results described in the included publications, sorted by primary target group and ascending education length.

Education primarily targeting managers, n = 6					
**Author(s), year, ref.**	**Education participants**	Education length*	**Key content**	**Educational methods**	**Reported results**
Doody et al., 2024 [44]	Managers (nursing and social care, n = 88), registered nurses (n = 9), social care worker (n = 3), clinical nurse specialists (n = 2)	<1 month	Leadership theories and styles Person-centredness	Experiential learning, reflective inquiry, internal and external facilitation.	Leadership practice outcomes Mechanisms of impact Perceived challenges
Mansell et al., 2008 [27]	Managers for intellectual disability residential homes (n = 36)	<1 month + workbased learning	Person-centredness	Training followed by work-based learning based on external facilitation.	Care practice outcomes
Eide et al., 2016 [33]	Middle managers (nurses) in municipal health and care services and non-profit organisations (n = 9)	1.5 months	Leadership theories and styles	Web-based person-centred education, including practice-based projects, reflection and external facilitation.	Leadership practice outcomes Care practice outcomes Mechanisms of impact Perceived challenges
Årestedt et al. 2024 [46] Hurtig et al. 2025 [47]	Healthcare managers (n = 6)	6 months	Person-centredness Facilitation skills	Two approaches: 1) External facilitation, including a toolkit sent to managers, 2) the same toolkit as group 1 + 2-day meeting, and monthly video conference with internal facilitators.	Care practice outcomes Perceived challenges
Wright and McCormack, 2001 [26]	Ward leader with a change facilitation role (n = 1)	12 months	Person-centredness Facilitation skills	Practice development grounded on external facilitation and action learning.	Care practice outcomes Mechanisms of impact
Jeon et al., 2015 [31]	Middle managers in residential and community aged care (n = 50)	12 months	Leadership theories and styles	Action learning grounded on external facilitation and practice-based improvement projects.	Leadership practice outcomes
**Education targeting people in non-managerial leadership roles, n = 8**					
**Author(s), year, ref.**	**Education participants**	Education length*	**Key content**	**Educational methods**	**Reported results**
Brooker et al., 2016 [32]	Dementia care coaches (nurses) with practice development facilitation roles (n = 66)	3 months + 6 supervision sessions	Person-centredness Facilitation skills	Practice development and a person-centred approach based on external and internal facilitation.	Leadership practice outcomes Care practice outcomes Mechanisms of impact Perceived challenges
McCormack et al., 2021 [41] Cable et al., 2024 [43]	Community nurses, nominated and selected to become Queen’s nurses (n = 20)	9 months	Leadership theories and styles Person-centredness Facilitation skills	Researcher-led workshops, masterclasses, active learning groups, paired and individual work, personal coaching, reflection assignment.	Leadership practice outcomes Mechanisms of impact
Bradd et al., 2018 [35]	Allied health professionals (n = 16)	10 months	Leadership theories and styles Facilitation skills	Action learning and individual coaching.	Leadership practice outcomes Care practice outcomes Mechanisms of impact
McCormack et al., 2022 [42]	Nursing and support staff (n = 70)	12 months	Person-centred cultures and practice Person-centredness	Workplace learning, action planning, participatory evaluation, and reflection, based on external facilitation.	Mechanisms of impact Perceived challenges
Stanhope et al., 2019 [40]	Mental health care providers with leadership or supervision roles (n = 40) and direct care staff (n = 64)	12 months	Person-centredness	In-person training, educational materials and a manual, followed by external facilitation by phone.	Care practice outcomes Perceived challenges
Hardiman and Dewing 2019 [39]	Nurses with clinical leadership and/or management roles (n = 5)	18 months	Person-centredness Facilitation skills	Workplace facilitation, shared learning, culture survey, and critical dialogue, based on external and internal facilitation.	Care practice outcomes Mechanisms of impact Perceived challenges
Manley and Titchen, 2017 [34]	Consultant nurses and midwives with clinical consultant roles (n = 10)	18 months	Facilitation skills	Action spirals, workshops, tools, 360-degree feedback and reflective review, based on external and internal facilitation.	Leadership practice outcomes Care practice outcomes Mechanisms of impact Perceived challenges
Boomer and McCormack 2010 [28]	Nurses with clinical leadership roles (n = 48)	36 months	Leadership theories and styles Person-centredness	Work-based learning utilising workshops, vision statements, action plans and action learning sets based on external and internal facilitation.	Leadership practice outcomes Care practice outcomes Mechanisms of impact Perceived challenges
**Education targeting mixed leadership groups (managers and leaders), n = 6**					
**Author(s), year, ref.**	**Education participants**	Education length*	**Key content**	**Educational methods**	**Reported results**
Dellenborg et al., 2019 [38]	Nurse assistants (n = 3), registered nurses (n = 8), and physicians (n = 12) all with formal or informal leadership roles, and management leaders (n = 2)	3 months	Person-centredness	External facilitation and interprofessional learning groups, formed by a practice-based steering committee.	Care practice outcomes Mechanisms of impact Perceived challenges
Lood et al., 2024 [45]	Healthcare professionals with formal or informal leadership roles (n = not applicable)	6 months	Leadership theories and styles Person-centredness	Externally facilitated lectures and workshops, practical home assignments, reflective practice, literature studies, and practice-based development projects.	Mechanisms of impact
Manley et al., 2014 [30]	Self-selecting specialist nurses, clinical leaders, practice development facilitators, consultant nurses, and site clinical managers (n = 400)	10 months	Leadership theories and styles Person-centredness Facilitation skills	Work-based learning, active learning sets, 360-degree feedback including patients and service users, and reflective review, based on external and internal facilitation.	Mechanisms of impact
Cardiff 2012 [29]	Unit manager (n = 1), charge nurses (n = 2), clinical nurse specialist (n = 1)	18 months	Leadership theories and styles Person-centredness	Critical and creative reflective inquiry through workshops, participant observations of context and leadership, and narratives of care and leadership, based on external facilitation.	Leadership practice outcomes Care practice outcomes Mechanisms of impact Perceived challenges
Cardiff et al., 2018 [32]	Unit nurse manager (n = 1), charge nurses (n = 2) clinical nurse (n = 1), primary nurses (n = 2)	36 months	Leadership theories and styles Person-centredness	Action spirals, workshops, observations, patient interviews, evaluation meetings, storytelling, and supervision, based on external and internal facilitation.	Leadership practice outcomes Care practice outcomes Mechanisms of impact Perceived challenges
Lynch et al. 2018 [37]	Aged care clinical leaders and managers (n = 6)	Not reported	Leadership theories and styles Person-centredness	Action learning spirals through creative and reflective dialogues and workshops.	Care practice outcomes Mechanisms of impact Perceived challenges

*Education length converted to months where necessary to enhance comparability.

### Key content

Key content was defined as the substantive topics, competencies, and frameworks addressed within leadership education. These are described in three broad categories: 1) *Leadership theories and styles*, 2) *Person-centredness*, and 3) *Facilitation skills*.

### Leadership theories and styles

Leadership theories and styles were identified as key content in 12 interventions and were used to frame leadership as a practice that enables cultural and practice change. Across these interventions, leadership was predominantly conceptualised as a relational, values-driven, and developmental practice. Five interventions focused explicitly on person-centred leadership, describing leadership as a dynamic and enabling process that empowers both leaders and direct care staff by nurturing trust, shared responsibility, and collaborative workplace cultures as prerequisites for person-centred care [29,31,36,37,45]. Person-centred leadership was conceptualised using several definitions. Cardiff [29] drew on Plas and Lewis [48], Cardiff et al. [36] applied McCormack and McCance’s framework [6], Lood et al. [45] used Eide and Cardiff’s definition [2], and Lynch et al. [37] referred to person-centred situational leadership as described by Lynch [49]. Jeon et al. [31] incorporated person- or client-centred leadership strategies operationalised as clinical leadership qualities [50]. Other leadership theories and styles included Theory U-based transformative leadership [51] in two interventions [41,43], transformational leadership based on Bass and Avolio [52] in three interventions [30,35,44], and ethical leadership drawing on multiple theoretical perspectives in one intervention [33]. In addition, transformational leadership was applied by Manley et al. [30], and reflective leadership by Boomer and McCormack [28], although without explicit theoretical referencing. One intervention further described leadership styles related to communication, empowerment, decision-making, operational management, and governance [44].

### Person-centredness

Person-centredness was identified as key content in 14 interventions and was conceptualised using multiple established frameworks addressing care, care planning, ethics, nursing, culture, leadership, and patient participation. Most commonly, person-centredness was framed as a relational approach emphasising collaboration, dignity, and mutual respect [28,29,36,37,39,41–43], drawing on McCormack and McCance’s Person-centred nursing framework [53] or the further developed Person-centred practice framework [6]. Four interventions applied the University of Gothenburg Centre for Person-Centred Care (GPCC) framework [7], emphasising person-centred ethics, leadership, and patient participation in recognising each persons’ resources and needs [38,45–47]. Other conceptualisations included the VIPS framework for dementia care [54], described in the intervention by Brooker et al. [32], person-centred care planning [40], tailored services [30], and frameworks for person-centred care planning in services for persons with disabilities [55]. Despite conceptual variation, all frameworks shared a common focus on partnership, recognition of personhood, and integration of person-centred values into leadership and organisational practices.

### Facilitation skills

Facilitation skills were identified as key content in eight interventions evaluated across 10 publications [26,30,32,34,35,39,41,43,46,47]. Facilitation skills referred to the concrete competencies to support learning, enable dialogue, and create conditions for change, collaboration and reflection. In the eight interventions, facilitation skills were positioned as central to workplace learning and implementation. In one publication, facilitation was described as supporting reflective practice, problem-solving, and professional growth, enabling direct care staff and leaders to take ownership of learning and change [34]. Two interventions [46,47] explicitly linked facilitation to implementation efforts through the integrated Promoting Action on Research Implementation in Health Services (i-PARIHS) framework [56], highlighting facilitation as support for systematic and context-sensitive change in health services [46,47]. Facilitation skills were further described as relational and developmental, supporting shared learning, collaboration, and transformation within teams [35,39,41], and as essential for cultivating person-centred relationships and practices [39]. In one intervention, facilitation skills were operationalised through coaching and role modelling, emphasising leaders’ roles in enacting person-centred ethics in everyday practice [32].

### Educational methods

Common educational methods included action learning [26,28,30,31,34–37,41,43], reflection [29,30,34,36,37,41–45], and practice-based learning [26–28,30–32,38,39,42,44–47]. All interventions utilised internal and/or external facilitation by researchers and/or appointed staff members. Additional methods included team-based learning, 360-degree feedback, coaching, and practice-based projects. Several interventions also employed participatory and emancipatory strategies to promote ethical leadership, interprofessional collaboration, and transformative change.

### Reported results

Reported results encompassed both experiential accounts and evaluated effects and were synthesised into four categories: 1) *Leadership practice outcomes*, 2) *Care practice outcomes*, 3), *Mechanisms of impact*, and 4) *Perceived challenges*.

### Leadership practice outcomes

Leadership practice outcomes primarily reflected changes in how participants enacted leadership following the interventions. Reported results included steps taken towards implementing knowledge gained through the interventions, that is, the “how” of leading towards person-centred care. Education participants reported improved skills in leading oneself and others, alongside greater sense of belonging and personal growth. They described finding their voice, creating safe spaces for engagement with co-workers and patients grounded in respect and a shared vision [35,43]. Reported outcomes included improved leadership effectiveness [29], relational capacity in relation to direct care staff and other leaders [29,33,36], reflexivity [33,34,36,41,43,44], heightened awareness of the importance of communication, person-centredness, advocacy, support, role modelling, and empowerment [44], and accessibility [28]. Leaders were also reported to have an increased ability to balance contextual demands with developmental needs [36], and more willing to experiment with innovative leadership approaches [32]. These outcomes were predominantly based on qualitative self-reports and reflective accounts.

### Care practice outcomes

Care practice outcomes related to perceived changes in care practices and workplace culture from the perspectives of education participants, healthcare staff, and patients. The most frequently reported outcome was a shift towards a more person-centred and learning-oriented culture [26–29,32,34–40]. This was described in terms of improved teamwork [28,36,38], more positive staff attitudes towards patients and improved knowledge of diagnoses [32], increased focus on relationships rather than tasks [36], improved continuity and coordination of care [32], and greater patient involvement in care and activities [26–28,35,38]. Quantitative evaluations were limited, and statistically significant effects on patient participation were not consistently demonstrated. Nevertheless, Hurtig et al. [47] and Årestedt et al. [46], reported increased awareness of attitudes and behaviours in patient encounters, perceived as supporting alignment with patients’ agendas [46,47]. Additional outcomes included increased motivation to challenge existing practices [26,33,35,36,38], more positive job experiences [35,36], reduced dependence on leaders, and higher engagement and energy levels among direct care staff [29].

### Mechanisms of impact

Mechanisms contributing to reported impact were most commonly described as structured reflection [26,29,33,35–39,41–44], active learning [26,28,29,33–35,38,39,45], a person-centred approach to learning [29,32,35–37,45], and timely feedback [30,33,39]. Reflection and reflective inquiry were identified as central to supporting leaders’ self-care and self-growth, with implications for community and practice-level impact [41]. Tailoring leadership education to specific professional groups, such as allied health leaders, was also reported to enhance the relevance and effectiveness of educational interventions [35].

### Perceived challenges

Perceived challenges highlighted limitations and tensions within leadership education for person-centred care. Education participants described relational challenges, including balancing leadership responsibilities with personal development and experiencing guilt relating to prioritising leadership learning [28]. They also found it more challenging than expected to remain focused on others and to ask appropriate questions at the right time to support the sharing their narratives [29,36,39]. Critically reflecting on one’s practice and letting go of traditional leadership and learning approaches was described as both challenging and beneficial [33,34,36,39,42]. Although reflective inquiry was generally viewed as valuable, some participants struggled with it, indicating a need for additional support in developing reflection skills [28]. Further challenges related to the complexity of facilitating implementation of person-centred care. While education participants valued the educational opportunities, changing workplace norms required more time and broader organisational engagement than anticipated [46]. Limited time for implementation and constrained ability to balance contextual demands with implementation needs were also reported [29,32,33,36–38,40,44,46]. Action learning was described as both enabling and constraining impact, depending on group composition, group dynamics, and the establishment of trust within learning groups [28,33,34,37].

### Knowledge gaps presented in the included publications

The knowledge gaps explicitly presented in the included publications primarily concerned the practical and organisational steps of implementing leadership education for person-centred care. These included limited evaluation of organisational readiness for change [40], restricted opportunities to apply learning in practice [27,35,38,39], challenges related to intervention fidelity [47], and methodological limitations such as cross-sectional designs and a lack of validated outcome measures [44]. Additional gaps concerned unclear educational objectives and roles [27,28,30,37–39], limited organisational willingness to invest in reflective learning beyond daily routines [44], and limited diversity among education participants. Patient and relative perspectives were underrepresented [31,36,44], as were staff beyond nursing, including allied health professionals [35], home care staff [32], and other healthcare roles [34]. Although many interventions adopted participatory approaches with healthcare professionals and staff, patient and relative involvement was infrequent and typically limited in scope. Suggested directions for future research included greater focus on the practical processes of implementing change [45], clarification of workplace roles in leadership education [32], and qualitative research and evaluations conducted in real-world practice contexts [37,39].

## Discussion

This scoping review mapped the existing evidence on leadership education designed to support person-centred care. Overall, the evidence base was small and unevenly distributed, with most educational interventions conducted in Northern Europe and predominantly involving non-managerial leaders, most often registered nurses. Interventions focused on leadership, person-centredness and facilitation, and the education was commonly delivered as longitudinal, work-based learning supported by facilitation, reflection, and active learning. Although reported outcomes were consistently positive, their interpretation is constrained by substantial methodological and contextual limitations affecting transferability and generalisability.

A key finding was the strong geographic concentration of studies in Northern Europe, reflecting a regional bias and a global evidence gap, particularly in diverse and underrepresented health and social care systems. As implementation of person-centred care is inherently context-dependent [11], findings must be interpreted in relation to systems in which the educational interventions were developed. As described by Damschroder et al. [57], context encompasses system-level prerequisites, organisational conditions, social and physical learning environments, and theoretical foundations [57], and leadership practices are known to vary across regions and countries [58,59]. More specifically, person-centred care is more widely embedded in tax-funded, publicly managed systems with strong policy alignment with person-centred care (e.g., Sweden, Norway, United Kingdom, and Ireland) than in insurance-based, mixed, or out-of-pocket model-based systems [60]. These conditions likely shape both the feasibility and design of leadership education and help explain the limited geographical variation observed in this review, underscoring the need for evidence from a wider range of health and social care systems.

Most interventions targeted healthcare staff in leadership roles, with limited attention to strategic managers’ responsibilities for enabling system-level transformation. This imbalance highlights challenges related to mandate and authority, identified by the reference group as critical for leaders seeking to drive care and culture change. As this review illustrates, significant challenges can arise when implementing person-centred care. Nilsen et al. [61] suggest that healthcare staff engaged in improving healthcare services must be prepared to lead change [61], and that support from management is critical when changing practices [62]. Considering that implementing person-centred care requires both resources and organisational structures [63], these findings point to a need for leadership education that explicitly addresses organisational- and system-level responsibilities, including resource allocation, quality governance, and strategic alignment, to support sustainable implementation.

Interprofessional inclusion was also limited, with most education participants in being registered nurses. While this may reflect workforce composition in certain settings, it risks reinforcing professional silos and undermining team-based care, as described by Cable et al. [43]. Evidence from clinical leadership education highlights the importance of interprofessional learning for collaboration and teamwork [64]. Effective person-centred care depends on strong interdisciplinary collaboration, and excluding allied health professionals, physicians, and social care staff may limit shared ownership of person-centred values and integrated practice [65]. Future leadership education should therefore explicitly support interprofessional collaboration across roles, organisations, and care settings.

Regarding key content, theoretical integration was weak in the included publications. Although some interventions applied specific leadership theories (e.g., Theory U-based transformative or person-centred leadership), many lacked explicit theoretical grounding. This should be interpreted in light of the broader leadership literature, which conceptualises leadership as a field characterised by theoretical diversity, conceptual overlap, and strong context dependency [66]. As described by Klinga et al. [67], overlap between leadership approaches is not inherently problematic [67], and leadership theories frequently share core elements, such as relationality, values, influence, and sense-making. Leadership approaches are often adapted pragmatically to specific organisational and cultural contexts rather than applied as discrete or mutually exclusive models [66,68]. However, within the included publications, theoretical plurality was rarely articulated or justified. Leadership styles, competencies, and theories were often used with limited clarification of their theoretical origins, distinctions, or intended mechanisms of action, making it difficult to determine how leadership education was expected to operate or which mechanisms supported change.

Person-centred care was similarly conceptualised using multiple frameworks, most commonly those developed by McCormack and McCance [6,53], emphasising relationships between leaders, direct care staff, and patients. However, few publications explicitly integrated person-centred care frameworks with leadership theories in the design, delivery, or evaluation of leadership education. As described by Deuling et al. [17], such lack of explicit linkage limits the understanding of how leaders are intended to support the ethical, relational, and organisational dimensions of person-centred care and constrains interpretation and cumulative knowledge development [17]. Importantly, the main limitation is not the use of multiple theories, but rather the absence of explicit theoretical integration and contextual justification. Future leadership education for person-centred care would therefore benefit from clearly articulating which theories are drawn upon, why they are appropriate for specific contexts, and how they are expected to support person-centred care. Such theoretical transparency is essential to strengthen conceptual clarity, enable meaningful comparison across studies, and support knowledge development in this field.

Overall, leadership education interventions were associated with reported improvements in leadership and care practices, but evidence of implementation outcomes and sustained system-wide impact was limited. Educational methods such as reflection, active learning, person-centred learning approaches, and timely feedback align with broader leadership education research [64]. Reflection is considered central to developing person-centred care and for understanding power dynamics in healthcare settings [6], though education participants reported challenges related to reflective skills, group dynamics, and trust. As noted by Philipson et al. [64], leadership education tailored to both individual and organisational needs tends to yield more favourable outcomes [64], yet leadership’s multifaceted nature complicates evaluation and comparison of leadership education across contexts [13] and contributes to the limited generalisability of the present findings.

Action research approaches were featured prominently among the included publications, illustrating how leadership education for person-centred care has largely been developed through relational, workplace-based inquiry. These approaches aim to support deep learning through close engagement with practice contexts and meaningful relationships, aligning well with the ethical and relational foundations of person-centred care. However, action research designs in the included publications were typically small-scale, long-term, and resource intensive, and education participants’ dual roles as co-researchers or practitioner-researchers sometimes blurred distinctions between educational participation and research involvement. Only one large-scale intervention was identified [30], highlighting a key limitation of the current evidence base. While action research offers valuable insights into learning processes and contextual change, its predominance constrains conclusions regarding scalability, comparative effectiveness, and system-wide impact. This underscores the need for leadership education that retains educational and relational depth, while being feasible to implement at scale, potentially through academic-practice partnerships.

Finally, this review highlights important gaps in understanding how leadership education translates into leadership practice, particularly with regard to the involvement of patients and relatives. Despite the centrality of partnership in person-centred care, such perspectives were often absent from the leadership education interventions, potentially reflecting the limited prioritisation of patient and public involvement at the time of publication, as described by Lu et al. [69]. Barriers to involvement, including hierarchical structures, paternalistic cultures, lack of recognition, discomfort, perceived effort, fear of disruption, representativeness issues, recruitment challenges, ethical concerns, resistance to change, and resource constraints, remain well documented [70–72]. Nevertheless, meaningful patient and public involvement has the potential to strengthen leadership education and align it more closely with the ethical foundations of person-centred are [7]. Together with the limited attention to implementation outcomes such as costs and resource implications, these gaps point to clear priorities for future research and development. Addressing them will be essential for advancing leadership education that can effectively and sustainably support the shift towards person-centred care.

### Strengths and limitations

This scoping review has several strengths. The inclusion of a reference group comprising patient and carer representatives, regional and municipal authorities, and professional organisations enhanced the relevance, credibility, and ethical sensitivity of the review through diverse perspectives and critical input throughout the process. Additional strengths include prospective protocol registration in the OSF, collaboration with an information specialist for the literature search, systematic screening and data extraction procedures, and an updated search that captured recently published studies. To minimise bias, the first and last authors, who co-authored one of the included publications, were not involved in screening, data extraction, or synthesis of that publication. The updated search further strengthened the review by capturing recently published studies. Several limitations should be considered when interpreting the findings. The included publications were highly heterogeneous in educational design, evaluation methods, and theoretical grounding, with many interventions having small samples, qualitative or action research designs, and non-comparative approaches. Few studies employed control groups, validated outcome measures, or longer-term follow-up. Consequently, reported positive outcomes should be interpreted as indications of perceived benefit and plausible mechanisms of change rather than evidence of effectiveness or causal impact, limiting conclusions regarding sustainability, scalability, and comparative effectiveness.

In line with scoping review methodology [21], no formal quality appraisal was conducted. While appropriate for mapping the evidence base, this further restricts interpretation for practice or policy recommendations. In addition, overlap between educator and researcher roles in several publications may have contributed to social desirability bias and underreporting of negative or unintended impact. The findings should therefore be regarded as exploratory and hypothesis-generating rather than confirmatory.

Conceptual and methodological challenges also affected the review. Variability in terminology related to leadership, person-centred care, and education may have resulted in the exclusion of relevant publications using alternative concepts. Although inclusion of the MeSH term “patient-centred care” might have increased retrieval, its exclusion was a deliberate conceptual choice, supported by literature distinguishing patient-centred care from person-centred care [4,23,73], and by evidence that dominant indexing terms can obscure conceptually distinct approaches in fields characterised by overlapping terminology [73]. Finally, restricting inclusion to publications in English, Finnish, Swedish, Norwegian, Danish or German and to indexed literature may have introduced language and publication bias. While necessary for feasibility and accurate interpretation, these restrictions may have limited the global representativeness of the findings. As with all literature reviews, publication bias cannot be fully eliminated, and caution is warranted when interpreting the results.

## Conclusions

This scoping review reveals a significant gap in the global evidence base on leadership education to support person-centred care. Of 2548 publications, only 22 met the inclusion criteria, indicating a narrow concentration of leadership concentration to Northern Europe and largely involving registered nurses in single-setting contexts. This limited scope reflects an uneven development of leadership education for person-centred care across health and social care systems. The findings suggest that leadership education grounded in longitudinal, work-based learning and incorporating reflective and participatory approaches may support leadership and care practices aligned with person-centred ethics. However, methodological limitations, including small sample sizes, lack of control groups, reliance on self-reported outcomes, and limited evaluation of implementation and system-level impact, mean that the findings should be interpreted as indicative rather than conclusive. To advance the field, leadership education should move beyond nursing- and Europe-centric models and engage a wider range of professional groups, leadership levels, and care contexts. Future interventions could for example include both frontline and strategic leaders, adopt interprofessional and inclusive approaches, and embed patient and relative perspectives to align with the ethical foundations of person-centred care. Strengthening theoretical integration between leadership and person-centred care frameworks, alongside more rigorous and transparent evaluation, will be essential.
