# Supplementary material for: Leadership education to support person-centred health and social care: A scoping review of empirical literature
Source: PLoS One. 2026 Jun 8;21(6):e0350991. doi: 10.1371/journal.pone.0350991 (PMC13245786; doi:10.1371/journal.pone.0350991)
Supplement: Attachment — Submitted filename: Response to reviewers.docx [file pone.0350991.s002.docx]

**Rebuttal letter**

*The authors would like to express their gratitude for the reviewers’ work and thoughtful comments. We have addressed the questions and revised the manuscript accordingly. We have also made some revisions in line with the editor’s comments below, added references, and revised the language in some sections of the manuscript. Our responses to the reviewers’ comments are provided below and all revisions are highlighted with yellow in the manuscript.*

Journal Requirements:

When submitting your revision, we need you to address these additional requirements.

1. Please ensure that your manuscript meets PLOS ONE's style requirements, including those for file naming. The PLOS ONE style templates can be found at <https://journals.plos.org/plosone/s/file?id=wjVg/PLOSOne_formatting_sample_main_body.pdf>  and <https://journals.plos.org/plosone/s/file?id=ba62/PLOSOne_formatting_sample_title_authors_affiliations.pdf>

   *We have revised the manuscript to ensure that it meets PLOS ONE’s style requirements.*
2. In this instance it seems there may be acceptable restrictions in place that prevent the public sharing of your minimal data. However, in line with our goal of ensuring long-term data availability to all interested researchers, PLOS’ Data Policy states that authors cannot be the sole named individuals responsible for ensuring data access ([http://journals.plos.org/plosone/s/data-availability#loc-acceptable-data-sharing-methods](http://track.editorialmanager.com/CL0/http:%2F%2Fjournals.plos.org%2Fplosone%2Fs%2Fdata-availability%23loc-acceptable-data-sharing-methods/1/010f0198a7aad034-09398a6b-a337-4a6d-9f66-e029c9fd8b31-000000/I8OqcIASOLlFHZDUOw5f9DAduk4lJ858hgvqmUWx6gc=224)).

   Data requests to a non-author institutional point of contact, such as a data access or ethics committee, helps guarantee long term stability and availability of data. Providing interested researchers with a durable point of contact ensures data will be accessible even if an author changes email addresses, institutions, or becomes unavailable to answer requests.

   Before we proceed with your manuscript, please also provide non-author contact information (phone/email/hyperlink) for a data access committee, ethics committee, or other institutional body to which data requests may be sent. If no institutional body is available to respond to requests for your minimal data, please consider if there any institutional representatives who did not collaborate in the study, and are not listed as authors on the manuscript, who would be able to hold the data and respond to external requests for data access? If so, please provide their contact information (i.e., email address). Please also provide details on how you will ensure persistent or long-term data storage and availability.

   *We have revised the Data availability statement to clarify that we have no data to share other than the data included in the report. See page 35, lines 548-551.*
3. If the reviewer comments include a recommendation to cite specific previously published works, please review and evaluate these publications to determine whether they are relevant and should be cited. There is no requirement to cite these works unless the editor has indicated otherwise.

   *N/A*

**Reviewer 1**

1. Abstract

•Could specify the type of empirical studies included (e.g., qualitative, quantitative, mixed methods).

*We have revised the abstract and added information on this under Results, see page 2, lines 24-46.*

•Lacks explicit mention of limitations.

*We have revised the abstract significantly, omitting sub-headings, and we now explicitly mention limitations in relation to our interpretation of conclusions that can be drawn from the study. See page 2, lines 33-39, and lines 40-41.*

2. Introduction

•Could incorporate more comprehensive background literature to contextualize the research gap.

*We have added text and references to contextualise the research gap and need for a scoping review in the end of the introduction on page 4, lines 74-77, and pages 4-5, lines 88-101.*

•Does not explicitly state the research hypotheses or specific research questions.

*As this manuscript presents a scoping review with the aim to map the existing literature and highlight gaps in research, we did not conduct any critical appraisal or synthesis of evidence to support or refute a specific claim by testing a specific hypothesis. Instead, the review was guided by research questions provided in the end of the introduction, page 5.*

3. Methods

Weaknesses:
•Lacks the exact search strings or detailed search strategies.

*The exact search strings and detailed search strategies are provided in table 1, page 7-8. We have clarified this on page 6, lines, 141-142, and in the table title on page 7, line 148.*

•Exclusion of certain languages introduces potential bias; rationale should be thoroughly justified or alternatives discussed.

*We agree and we have stated the rationale behind our decision in the Methods section, page 8, lines 161-162. We have also elaborated on this in the Discussion under Strengths and limitations on page 34, lines 524-526.*

4. Results

•Insufficient detail on the nature and content of the educational interventions.

*We appreciate your perspective and understand the importance of clarity in this area. We have added information on the nature of the educational interventions on page 16, lines 223-230, and in table 3 on pages 17-22. With regards to education content, we believe that the current level of detail is appropriate for the scope of a scoping review, which prioritises mapping the evidence rather than evaluating individual interventions in depth. Nevertheless, we remain open to suggestions on how to improve clarity without compromising the overall structure and focus of the paper.*

•No assessment or discussion of the quality or risk of bias of included studies, which is acceptable for scoping reviews but should be acknowledged.

*We have elaborated on this in the Discussion under Strengths and limitations on page 33, lines 506-510.*

•Limited discussion of heterogeneity among studies and implications for evidence synthesis.

*Thank you for this observation. As noted in our previous response, our aim was not to synthesise universally generalisable evidence, but rather to map evidence and highlight knowledge gaps. We acknowledge the heterogeneity among the included publications and have elaborated on this in the abstract on page 2, lines 37-39. We have also expanded the discussion to reflect how this variation limits comparability of results between publications, while also pointing to important areas for future research. See page 31, lines 462-467.*

5. Discussion

•Does not critically evaluate the limitations of their review (e.g., language bias, small sample size).

*We have elaborated on the risk of language bias in the Discussion under Strengths and limitations on page 34, lines 524-526. We also discuss the implications of the small sample sizes in the included publications on page 33, lines 501-510.*

•Does not explore contextual factors (e.g., healthcare system differences abroad) that influence generalization.

*While contextual factors such as healthcare system differences are indeed relevant, they fall outside the scope of our research questions and were therefore not included in the results. However, we have added a note in the discussion acknowledging that healthcare systems can influence the adoption of person-centred care. Page 29, lines 400-404.*

6. Conclusions

• Slightly vague; specific actionable recommendations could strengthen the conclusion.
• Should explicitly state the limitations impacting interpretation.

*We have made significant revisions to the conclusion to provide more actionable recommendations, taking into account the limitations of the review. See page 34-35, lines 531-546.*

7. References

•Update and standardize all references to meet journal formatting guidelines.
•Ensure completeness with full bibliographic details.
•Include DOIs to improve accessibility.
•Maintain consistency in author listing, journal abbreviations, and sequential ordering.
 *Done*

•Consider adding additional recent empirical studies directly related to leadership education outcomes to strengthen the literature base.

*We have added the following references:*

- Parry K, Bryman A. Leadership in organizations. In: Clegg S, Hardy C, Lawrence T, Nord W, editors. The SAGE handbook of organization studies. London: SAGE Publ; 2013.
- Guibert-Lacasa C, Vázquez-Calatayud M. Nurses’ clinical leadership in the hospital setting: A systematic review. Journal of Nursing Management. 2022;30:913-25.
- Sparr J, Knipfer K, Willems F. How leaders can get the most out of formal training: The significance of feedback‐seeking and reflection as informal learning behaviors. Human Resource Development Quarterly. 2017;28(1):29-54.
- Phillipson J, Pinto AC, Kingsley-Smith H, Krachler N, McGivern G, Lyons O. Leadership training in healthcare: a systematic umbrella review. BMJ Lead. 2025.

Reviewer #2: It was a pleasure reading this article. The article is overall well written and tells an interesting story of a relevant issue.

*Thank you!*

Main points:
I would welcome if the abstract would foreshadow the type of criteria used for inclusion.

*We have added information on this in the abstract on page 2, lines 28-30.*

The concepts around Person centred care are well described in the introduction. The concept of leadership education could however be stronger introduced to follow more logically towards the research question. You could map out the so what question of your research more in the end of the introduction.

*We appreciate this insight, and we have added information on leadership on page 4, lines 74-77, and tried to map out the rationale for the review on pages 4-5, lines- 88-101.*

Personally I would move the statement on the term 'stakeholders' to the methods.

*We have now revised and moved this statement to the methods section under Data extraction process, pages 9-10, lines 188-190.*

In the methods, I miss a more thorough description of the data analysis, e.g. how was it performed, who was involved, how were the presented categories decided upon.

*We have elaborated on the analysis, providing information on how we performed the analysis (rather than synthesis), who was involved, and how the presented categories were decided upon to avoid interpretive synthesis. Please see page 10, lines 192-200.*

The search strategy is sound and I applaud the inclusion of an information scientist. My only question is why only formal activities were included in the search. The lack of informal learning activities should be addressed in the discussion, or you should consider adding this to your search.

*We appreciate your recognition of the involvement of an information scientist in developing the search strategy. While our research questions focused on education, which primarily highlights formal learning activities, we acknowledge the importance of informal learning in leadership development. A clarifying sentence has been added in the introduction (page 4, lines 90–93). We have also discussed how our definition of education may influence the searches on page 33, lines 515–517. The search strategy was carefully constructed to capture both formal education and informal learning activities relevant to person-centred care. For example, terms such as (MH learning+), (TI change OR AB change), and (TI implement* OR AB implement*) were included to reflect informal learning following leadership programmes.*

The search strategy seems to be limited to English language which presents a misalignment with what you describe in the text.

*Thank you for this comment which highlights a risk for misinterpretation in our reporting of the detailed search strategy. After discussing this with the information specialist involved in the search for literature, we have added information as part of Table 1 on pages 7-8, and we have added a table footnote on page 8, lines 149-150. As a clarification, we have also added the rationale for this decision on page 8, lines-161-162.*

I very much appreciate the overview given in Table 2

*Thank you!*

Results are overall well described. I would however move the part on Knowledge gaps and future research to the Discussion or highlight more neatly which is part of your own analysis and conclusion and what has been suggested by the included articles. Currently the findings seems too intertwined with the areas for future research.

*Thank you, we have revised the heading for this section of the results, to clarify that the knowledge gaps presented there are the ones presented in the included publications, see page 27, lin 360. We have also removed the last sentence: ”This highlights the need for more inclusive research that incorporates these perspectives.” and incorporated it in the discussion as that reflects our interpretation of the findings.*

While the discussion addresses relevant points, i would suggest having another critical look at how you can structure it better so that the summary of findings, their reflecting in light of current research and educational practice aligns and what practical and scientific implications you see.

*Thank you for this advice, we have revised the discussion with an introduction to the structure of the discussion (page 28, lines 384-387) and we have made revisions to clarify the findings in relation to current research and educational practice, see text highlighted in yellow in the results discussion on pages 28-32. The conclusion has been significantly revised to clarify the practical and scientific implications, pages 34-35, lines531-546.*

Minor points:
There is a small typo in the abstract under Setting, where education should be listed in singular instead of plural.

*We have corrected this.*
