# Supplementary material for: Leadership education to support person-centred health and social care: A scoping review of empirical literature
Source: PLoS One. 2026 Jun 8;21(6):e0350991. doi: 10.1371/journal.pone.0350991 (PMC13245786; doi:10.1371/journal.pone.0350991)
Supplement: Attachment — Submitted filename: Rebuttal letter R2.docx [file pone.0350991.s003.docx]

*The authors would like to express their gratitude for the reviewer’s work and thoughtful comments. We have revised the manuscript extensively in response to all comments, and we believe that these revisions have substantially strengthened the clarity, coherence, and scientific rigour of the paper. Below, we respond to each comment in turn. Major changes made to the manuscript are highlighted in yellow.*

Reviewer #3: Title and Keywords
• Title length: The full title is overly long and could be more concise while retaining clarity.
*The title has been retained as originally submitted, as it corresponds to the title registered in the review protocol and is consistent with recommended reporting guidelines. The title also falls well below the maximum length permitted by the journal. For these reasons, we have chosen not to modify the title.*

• Keywords: Some keywords (e.g., “manager”) are too generic and do not reflect the scope of the review; missing terms like “leadership training” or “scoping review.”
*We have revised the keywords accordingly. The term “manager” has been removed and “leadership training” has been added. We did not add “scoping review” as a keyword, as that is clearly stated in the title.*

Abstract
• Lack of clarity on study type: The abstract does not clearly specify the types of empirical studies included (qualitative, quantitative, mixed methods).

*The abstract has been revised to explicitly state that qualitative, quantitative, and mixed-methods empirical studies were eligible for inclusion, see page 2, lines 33-34.*

• Insufficient mention of limitations: While limitations are mentioned, they are vague and not linked to interpretation of findings.
*We have revised the abstract to provide a clearer and more explicit description of key limitations and to link these directly to the interpretation of the findings. See page 2, lines 42-47.*

• Overly descriptive: The abstract reads more like a summary than a structured scientific abstract; lacks concise statements on methodology and implications.

*The abstract has been substantially revised to follow a more scientific format, we hope you find it more structured and concise. See pages 2-23.*

• Typographical error: “educations” should be singular (“education”) under Setting.

*Thank you for noting this. The terminology has been corrected throughout the manuscript to education or education interventions, as appropriate.*

Introduction
• Insufficient theoretical framing: Leadership education is not introduced strongly enough; the link between person-centred care and leadership education lacks logical flow.

*The introduction has been revised to strengthen the theoretical framing of leadership and to improve the logical flow between person-centred care, leadership, and leadership education. See pages 3-5, lines 61-64, 70-72, 77-79, 82-88, 90-102, and 111-119.*

• Missing research questions upfront: Research questions appear late; hypotheses are absent (though scoping reviews typically do not test hypotheses, this should be clarified earlier).

*We have clarified the rationale for a scoping review approach on page 6, lines 126-128, for which hypothesis testing is not appropriate. The research questions are presented following the stated aim, in accordance with scoping review methodology.*

• Contextual gap: Limited discussion of global healthcare system differences affecting generalizability.

*We have expanded the introduction to include healthcare system variation and its implications for leadership education. See, pages 5-6, lines 121-125.*

Methods
• Search strategy reporting: Although Table 1 includes search strings, the rationale for excluding “patient-centred care” MeSH term is weak and could bias results.

*We have strengthened the justification for excluding this term in the Methods section (page 8, lines186-191) and further elaborated on the conceptual distinction between patient-centred and person-centred care in the introduction (page 3-4, lines 66-79) and we discuss it on pages 33-34, lines 574-578*

• Language bias: Restriction to certain languages introduces bias; justification is minimal.

*The rationale for language restrictions has been clarified in the methods (page 9, lines 175-177), and the implications of potential language bias are now explicitly addressed in the discussion, see page 34, lines 578-581.*

• Data analysis description: The process for coding and category development is underexplained; lacks detail on inter-rater reliability or validation.

*The Data analysis section has been revised to provide a clearer description of the process, including iterative category development, and validation procedures, see page 11. We acknowledge the reviewer’s comment regarding inter-rater reliability. In line with JBI guidance and qualitative methodology, we ensured rigour through triangulation and consensus-based category development, which we have now clarified in the Data analysis on page 11.*

• Interest holder definition: The term “interest holder” is introduced late and should be clarified earlier in Methods.

*The definition of interest holder is clarified in the methods section, and its use is consistent throughout the manuscript.*

Results
• Insufficient detail on interventions: Descriptions of educational interventions are brief and lack depth (e.g., pedagogical strategies, theoretical underpinnings).

*The results section has been substantially revised and restructured to provide more detailed and analytically organised descriptions of key content, educational methods, reported results, and knowledge gaps.*

• Heterogeneity not addressed: Wide variation in study designs and contexts is noted but not analyzed for implications.

*We now explicitly address heterogeneity in study design, context, and intervention characteristics and discuss its implications for interpretation and transferability in the Discussion section on pages 28-31, lines 431-447, 513-525, and in the Strengths and limitations on page 33, lines 552-559 and 563-566.*

• Tables: Table 3 is dense and lacks clarity; inconsistent formatting of education length and participant details.

*Table 3 has been revised for clarity and consistency. A streamlined version is now included in the main manuscript, while more details have been added to the text describing the results. In line with this comment, we have also revised table 2, omitting details on study participants as this may confuse the readers. our focus is on education participants (i.e., leaders), whereas study participants sometimes included direct care staff and patients.*

Discussion
• Weak critical analysis: Discussion does not sufficiently critique methodological limitations (e.g., small sample sizes, lack of control groups).

*We have strengthened the critical analysis of methodological limitations, including small sample sizes, lack of control groups, reliance on self-reported outcomes, and implications for interpretation (see revised discussion and strengths and limitations sections). See Strengths and limitations on pages 32-34.*

• Contextual factors missing: No exploration of how healthcare system differences influence applicability.

*We have expanded both the introduction and discussion to include healthcare system differences and their influence on applicability and generalisability. See for example pages 5-6, lines 121-125, and page 28, lines 435-447.*

• Integration with existing literature: Limited synthesis with broader leadership education research; theoretical integration is weak.

*The discussion has been revised to more explicitly integrate findings with broader leadership education and leadership theory literature, including recent work on leadership plurality, overlap between leadership approaches, context dependency, and co-leadership in integrated care settings. For example pages 29-30, lines 471-484 and the reference lists for updated references.*

Conclusions
• Vague recommendations: Conclusions are broad and lack actionable steps for educators or policymakers.

*The conclusions section has been substantially revised to provide clearer and more actionable implications for educators, researchers, and policymakers. See pages 34-35.*

• Limitations not linked to interpretation: While limitations are mentioned, their impact on findings is not clearly articulated.

*We now explicitly link key limitations to interpretation of findings both in the Strengths and Limitations and Conclusions sections.*

References
• Formatting inconsistencies: Some references lack proper DOI formatting; inconsistent use of italics and punctuation.

*All references have been carefully reviewed to ensure full compliance with formatting requirements.*

• Currency of literature: Although recent references were added, some seminal works on leadership education are missing.

*Additional recent and relevant literature on leadership education and leadership theory has been incorporated to strengthen the theoretical integration of the manuscript, informed by scholarly consultation acknowledged in the paper.*

• Redundancy: Multiple references from the same authors without clear justification for inclusion.

*We have reviewed the reference list to ensure that multiple citations from the same authors are justified by distinct contributions.*

9. Figures and Tables
• Figure 1 (PRISMA): Poor resolution and unclear labeling; does not meet journal quality standards.

*Figure 1 has been revised to improve resolution and labelling in accordance with journal standards.*

• Tables: Overly complex, lack clear headings, and inconsistent terminology (e.g., “education participants” vs. “study participants”).

*The tables have been revised to improve clarity, simplify structure, and ensure consistent terminology throughout (e.g., standardising the use of education participants and excluding information on study participants in table 2, as explained in our previous reply). Headings have been clarified and table content streamlined to enhance readability and alignment with the manuscript text.*

Language and Style
• Typographical errors: Examples include “Northen Europe” instead of “Northern Europe.”

*We hope that all typographical errors have been corrected.*

• Overuse of passive voice: Reduces clarity and engagement.

*The manuscript has undergone careful language revision to reduce unnecessary passive constructions and repetitive phrasing, improving readability and flow.*

• Repetitive phrasing: Frequent repetition of “person-centred care” without variation; could be condensed for readability.

*We acknowledge the reviewer’s comment. Given that person-centred care is the core concept of the manuscript and central to its theoretical and empirical focus, we have retained consistent terminology to ensure conceptual clarity and avoid ambiguity. Where possible, minor wording adjustments have been made to improve flow without substituting the term in ways that could alter its meaning.*

• Journal style compliance: Some sections (e.g., headings, abbreviations) do not fully align with PLOS ONE formatting guidelines.

*All sections, headings, abbreviations, and formatting have been reviewed and adjusted to align with submission guidelines.*

*We thank the reviewer again for the constructive feedback, which has significantly improved the quality and clarity of the manuscript.*
