# Supplementary material for: Leadership education to support person-centred health and social care: A scoping review of empirical literature
Source: PLoS One. 2026 Jun 8;21(6):e0350991. doi: 10.1371/journal.pone.0350991 (PMC13245786; doi:10.1371/journal.pone.0350991)
Supplement: Peer review history

# Peer review: Decision Letter 0

**DOI:** 10.1371/journal.pone.0350991.r001

**Authors:** Amal Diab Ghanem Atalla

14 Aug 2025

PONE-D-25-20590

Leadership education to support person-centred health and social care: a scoping review of empirical literature

PLOS ONE

Dear Dr. Lood,

Thank you for submitting your manuscript to PLOS ONE. After careful consideration, we feel that it has merit but does not fully meet PLOS ONE’s publication criteria as it currently stands. Therefore, we invite you to submit a revised version of the manuscript that addresses the points raised during the review process.

==============================

**ACADEMIC EDITOR:**

Dear Author,

Thank you for submitting your manuscript to our Journal. After careful evaluation by our reviewers and the editorial team, we have determined that your manuscript has merit but requires major revisions before it can be considered for publication. The reviewers have identified several key areas that need significant improvement, including [briefly mention the main concerns, e.g., methodological limitations, insufficient data analysis, unclear presentation of results, or inadequate discussion]. We encourage you to carefully address each of the reviewers' comments and provide a detailed response outlining the changes made. If any suggestions cannot be implemented, please provide a clear justification. Given the extent of the required revisions, your manuscript will undergo another round of peer review upon resubmission. Please ensure that your revised manuscript adheres to the journal’s formatting and reporting guidelines. We appreciate your efforts and look forward to receiving your revised manuscript. Please do not hesitate to reach out if you require any clarification.

Best regards,

[Amal Diab Ghanem Atalla]

[Academic editor]

==============================

Please submit your revised manuscript by Sep 27 2025 11:59PM. If you will need more time than this to complete your revisions, please reply to this message or contact the journal office at plosone@plos.org. When you're ready to submit your revision, log on to https://www.editorialmanager.com/pone/ and select the 'Submissions Needing Revision' folder to locate your manuscript file.

Please include the following items when submitting your revised manuscript:

- A rebuttal letter that responds to each point raised by the academic editor and reviewer(s). You should upload this letter as a separate file labeled 'Response to Reviewers'.
- A marked-up copy of your manuscript that highlights changes made to the original version. You should upload this as a separate file labeled 'Revised Manuscript with Track Changes'.
- An unmarked version of your revised paper without tracked changes. You should upload this as a separate file labeled 'Manuscript'.

If you would like to make changes to your financial disclosure, please include your updated statement in your cover letter. Guidelines for resubmitting your figure files are available below the reviewer comments at the end of this letter.

If applicable, we recommend that you deposit your laboratory protocols in protocols.io to enhance the reproducibility of your results. Protocols.io assigns your protocol its own identifier (DOI) so that it can be cited independently in the future. For instructions see: https://journals.plos.org/plosone/s/submission-guidelines#loc-laboratory-protocols. Additionally, PLOS ONE offers an option for publishing peer-reviewed Lab Protocol articles, which describe protocols hosted on protocols.io. Read more information on sharing protocols at https://plos.org/protocols?utm_medium=editorial-email&utm_source=authorletters&utm_campaign=protocols.

We look forward to receiving your revised manuscript.

Kind regards,

Amal Diab Ghanem Atalla

Academic Editor

PLOS ONE

Journal Requirements:

When submitting your revision, we need you to address these additional requirements.

1. Please ensure that your manuscript meets PLOS ONE's style requirements, including those for file naming. The PLOS ONE style templates can be found at

https://journals.plos.org/plosone/s/file?id=wjVg/PLOSOne_formatting_sample_main_body.pdf  and

https://journals.plos.org/plosone/s/file?id=ba62/PLOSOne_formatting_sample_title_authors_affiliations.pdf

2. In this instance it seems there may be acceptable restrictions in place that prevent the public sharing of your minimal data. However, in line with our goal of ensuring long-term data availability to all interested researchers, PLOS’ Data Policy states that authors cannot be the sole named individuals responsible for ensuring data access (http://journals.plos.org/plosone/s/data-availability#loc-acceptable-data-sharing-methods).

Data requests to a non-author institutional point of contact, such as a data access or ethics committee, helps guarantee long term stability and availability of data. Providing interested researchers with a durable point of contact ensures data will be accessible even if an author changes email addresses, institutions, or becomes unavailable to answer requests.

Before we proceed with your manuscript, please also provide non-author contact information (phone/email/hyperlink) for a data access committee, ethics committee, or other institutional body to which data requests may be sent. If no institutional body is available to respond to requests for your minimal data, please consider if there any institutional representatives who did not collaborate in the study, and are not listed as authors on the manuscript, who would be able to hold the data and respond to external requests for data access? If so, please provide their contact information (i.e., email address). Please also provide details on how you will ensure persistent or long-term data storage and availability.

3. If the reviewer comments include a recommendation to cite specific previously published works, please review and evaluate these publications to determine whether they are relevant and should be cited. There is no requirement to cite these works unless the editor has indicated otherwise.

Additional Editor Comments:

Dear Author,

Thank you for submitting your manuscript to our Journal. After careful evaluation by our reviewers and the editorial team, we have determined that your manuscript has merit but requires major revisions before it can be considered for publication.

The reviewers have identified several key areas that need significant improvement, including [briefly mention the main concerns, e.g., methodological limitations, insufficient data analysis, unclear presentation of results, or inadequate discussion]. We encourage you to carefully address each of the reviewers' comments and provide a detailed response outlining the changes made. If any suggestions cannot be implemented, please provide a clear justification.

Given the extent of the required revisions, your manuscript will undergo another round of peer review upon resubmission. Please ensure that your revised manuscript adheres to the journal’s formatting and reporting guidelines.

We appreciate your efforts and look forward to receiving your revised manuscript. Please do not hesitate to reach out if you require any clarification.

Best regards,

[Amal Diab Ghanem Atalla]

[Academic editor]

[Note: HTML markup is below. Please do not edit.]

Reviewers' comments:

Reviewer's Responses to Questions

**Comments to the Author**

1. Is the manuscript technically sound, and do the data support the conclusions?

The manuscript must describe a technically sound piece of scientific research with data that supports the conclusions. Experiments must have been conducted rigorously, with appropriate controls, replication, and sample sizes. The conclusions must be drawn appropriately based on the data presented.

Reviewer #1: Yes

Reviewer #2: Yes

**********

2. Has the statistical analysis been performed appropriately and rigorously?

Reviewer #1: N/A

Reviewer #2: N/A

**********

3. Have the authors made all data underlying the findings in their manuscript fully available?

The PLOS Data policy requires authors to make all data underlying the findings described in their manuscript fully available without restriction, with rare exception (please refer to the Data Availability Statement in the manuscript PDF file). The data should be provided as part of the manuscript or its supporting information, or deposited to a public repository. For example, in addition to summary statistics, the data points behind means, medians and variance measures should be available. If there are restrictions on publicly sharing data—e.g. participant privacy or use of data from a third party—those must be specified.

Reviewer #1: Yes

Reviewer #2: Yes

**********

4. Is the manuscript presented in an intelligible fashion and written in standard English?

PLOS ONE does not copyedit accepted manuscripts, so the language in submitted articles must be clear, correct, and unambiguous. Any typographical or grammatical errors should be corrected at revision, so please note any specific errors here.

Reviewer #1: Yes

Reviewer #2: Yes

**********

5. Review Comments to the Author

Please use the space provided to explain your answers to the questions above. You may also include additional comments for the author, including concerns about dual publication, research ethics, or publication ethics. (Please upload your review as an attachment if it exceeds 20,000 characters)

Reviewer #1: 1. Abstract

•Could specify the type of empirical studies included (e.g., qualitative, quantitative, mixed methods).

•Lacks explicit mention of limitations.

2. Introduction

•Could incorporate more comprehensive background literature to contextualize the research gap.

•Does not explicitly state the research hypotheses or specific research questions.

3. Methods

Weaknesses:

•Lacks the exact search strings or detailed search strategies.

•Exclusion of certain languages introduces potential bias; rationale should be thoroughly justified or alternatives discussed.

4. Results

•Insufficient detail on the nature and content of the educational interventions.

•No assessment or discussion of the quality or risk of bias of included studies, which is acceptable for scoping reviews but should be acknowledged.

•Limited discussion of heterogeneity among studies and implications for evidence synthesis.

5. Discussion

•Does not critically evaluate the limitations of their review (e.g., language bias, small sample size).

•Does not explore contextual factors (e.g., healthcare system differences abroad) that influence generalization.

6. Conclusions

Slightly vague; specific actionable recommendations could strengthen the conclusion.

Should explicitly state the limitations impacting interpretation.

7. References

•Update and standardize all references to meet journal formatting guidelines.

•Ensure completeness with full bibliographic details.

•Include DOIs to improve accessibility.

•Maintain consistency in author listing, journal abbreviations, and sequential ordering.

•Consider adding additional recent empirical studies directly related to leadership education outcomes to strengthen the literature base.

Reviewer #2: It was a pleasure reading this article. The article is overall well written and tells an interesting story of a relevant issue.

Main points:

I would welcome if the abstract would foreshadow the type of criteria used for inclusion.

The concepts around Person centred care are well described in the introduction. The concept of leadership education could however be stronger introduced to follow more logically towards the research question. You could map out the so what question of your research more in the end of the introduction.

Personally I would move the statement on the term 'stakeholders' to the methods.

In the methods, I miss a more thorough description of the data analysis, e.g. how was it performed, who was involved, how were the presented categories decided upon. The search strategy is sound and I applaud the inclusion of an information scientist. My only question is why only formal activities were included in the search. The lack of informal learning activities should be addressed in the discussion, or you should consider adding this to your search.

The search strategy seems to be limited to English language which presents a misalignment with what you describe in the text.

I very much appreciate the overview given in Table 2

Results are overall well described. I would however move the part on Knowledge gaps and future research to the Discussion or highlight more neatly which is part of your own analysis and conclusion and what has been suggested by the included articles. Currently the findings seems too intertwined with the areas for future research.

While the discussion addresses relevant points, i would suggest having another critical look at how you can structure it better so that the summary of findings, their reflecting in light of current research and educational practice aligns and what practical and scientific implications you see.

Minor points:

There is a small typo in the abstract under Setting, where education should be listed in singular instead of plural.

**********

6. PLOS authors have the option to publish the peer review history of their article (what does this mean?). If published, this will include your full peer review and any attached files.

If you choose “no”, your identity will remain anonymous but your review may still be made public.

**Do you want your identity to be public for this peer review?** For information about this choice, including consent withdrawal, please see our Privacy Policy.

Reviewer #1: No

Reviewer #2: No

**********

[NOTE: If reviewer comments were submitted as an attachment file, they will be attached to this email and accessible via the submission site. Please log into your account, locate the manuscript record, and check for the action link "View Attachments". If this link does not appear, there are no attachment files.]

While revising your submission, please upload your figure files to the Preflight Analysis and Conversion Engine (PACE) digital diagnostic tool, https://pacev2.apexcovantage.com/. PACE helps ensure that figures meet PLOS requirements. To use PACE, you must first register as a user. Registration is free. Then, login and navigate to the UPLOAD tab, where you will find detailed instructions on how to use the tool. If you encounter any issues or have any questions when using PACE, please email PLOS at figures@plos.org. Please note that Supporting Information files do not need this step.

# Author comment: Author response to Decision Letter 1

**DOI:** 10.1371/journal.pone.0350991.r002

1 Oct 2025

The authors would like to express their gratitude for the reviewers’ work and thoughtful comments. We have addressed the questions and revised the manuscript accordingly. We have also made some revisions in line with the editor’s comments below, added references, and revised the language in some sections of the manuscript. Our responses to the reviewers’ comments are provided below and all revisions are highlighted with yellow in the manuscript.

Journal Requirements:

When submitting your revision, we need you to address these additional requirements.

1. Please ensure that your manuscript meets PLOS ONE's style requirements, including those for file naming. The PLOS ONE style templates can be found at https://journals.plos.org/plosone/s/file?id=wjVg/PLOSOne_formatting_sample_main_body.pdf and https://journals.plos.org/plosone/s/file?id=ba62/PLOSOne_formatting_sample_title_authors_affiliations.pdf

We have revised the manuscript to ensure that it meets PLOS ONE’s style requirements.

2. In this instance it seems there may be acceptable restrictions in place that prevent the public sharing of your minimal data. However, in line with our goal of ensuring long-term data availability to all interested researchers, PLOS’ Data Policy states that authors cannot be the sole named individuals responsible for ensuring data access (http://journals.plos.org/plosone/s/data-availability#loc-acceptable-data-sharing-methods).

Data requests to a non-author institutional point of contact, such as a data access or ethics committee, helps guarantee long term stability and availability of data. Providing interested researchers with a durable point of contact ensures data will be accessible even if an author changes email addresses, institutions, or becomes unavailable to answer requests.

Before we proceed with your manuscript, please also provide non-author contact information (phone/email/hyperlink) for a data access committee, ethics committee, or other institutional body to which data requests may be sent. If no institutional body is available to respond to requests for your minimal data, please consider if there any institutional representatives who did not collaborate in the study, and are not listed as authors on the manuscript, who would be able to hold the data and respond to external requests for data access? If so, please provide their contact information (i.e., email address). Please also provide details on how you will ensure persistent or long-term data storage and availability.

We have revised the Data availability statement to clarify that we have no data to share other than the data included in the report. See page 35, lines 548-551.

3. If the reviewer comments include a recommendation to cite specific previously published works, please review and evaluate these publications to determine whether they are relevant and should be cited. There is no requirement to cite these works unless the editor has indicated otherwise.

N/A

Reviewer 1

1. Abstract

•Could specify the type of empirical studies included (e.g., qualitative, quantitative, mixed methods).

We have revised the abstract and added information on this under Results, see page 2, lines 24-46.

•Lacks explicit mention of limitations.

We have revised the abstract significantly, omitting sub-headings, and we now explicitly mention limitations in relation to our interpretation of conclusions that can be drawn from the study. See page 2, lines 33-39, and lines 40-41.

2. Introduction

•Could incorporate more comprehensive background literature to contextualize the research gap.

We have added text and references to contextualise the research gap and need for a scoping review in the end of the introduction on page 4, lines 74-77, and pages 4-5, lines 88-101.

•Does not explicitly state the research hypotheses or specific research questions.

As this manuscript presents a scoping review with the aim to map the existing literature and highlight gaps in research, we did not conduct any critical appraisal or synthesis of evidence to support or refute a specific claim by testing a specific hypothesis. Instead, the review was guided by research questions provided in the end of the introduction, page 5.

3. Methods

Weaknesses:

•Lacks the exact search strings or detailed search strategies.

The exact search strings and detailed search strategies are provided in table 1, page 7-8. We have clarified this on page 6, lines, 141-142, and in the table title on page 7, line 148.

•Exclusion of certain languages introduces potential bias; rationale should be thoroughly justified or alternatives discussed.

We agree and we have stated the rationale behind our decision in the Methods section, page 8, lines 161-162. We have also elaborated on this in the Discussion under Strengths and limitations on page 34, lines 524-526.

4. Results

•Insufficient detail on the nature and content of the educational interventions.

We appreciate your perspective and understand the importance of clarity in this area. We have added information on the nature of the educational interventions on page 16, lines 223-230, and in table 3 on pages 17-22. With regards to education content, we believe that the current level of detail is appropriate for the scope of a scoping review, which prioritises mapping the evidence rather than evaluating individual interventions in depth. Nevertheless, we remain open to suggestions on how to improve clarity without compromising the overall structure and focus of the paper.

•No assessment or discussion of the quality or risk of bias of included studies, which is acceptable for scoping reviews but should be acknowledged.

We have elaborated on this in the Discussion under Strengths and limitations on page 33, lines 506-510.

•Limited discussion of heterogeneity among studies and implications for evidence synthesis.

Thank you for this observation. As noted in our previous response, our aim was not to synthesise universally generalisable evidence, but rather to map evidence and highlight knowledge gaps. We acknowledge the heterogeneity among the included publications and have elaborated on this in the abstract on page 2, lines 37-39. We have also expanded the discussion to reflect how this variation limits comparability of results between publications, while also pointing to important areas for future research. See page 31, lines 462-467.

5. Discussion

•Does not critically evaluate the limitations of their review (e.g., language bias, small sample size).

We have elaborated on the risk of language bias in the Discussion under Strengths and limitations on page 34, lines 524-526. We also discuss the implications of the small sample sizes in the included publications on page 33, lines 501-510.

•Does not explore contextual factors (e.g., healthcare system differences abroad) that influence generalization.

While contextual factors such as healthcare system differences are indeed relevant, they fall outside the scope of our research questions and were therefore not included in the results. However, we have added a note in the discussion acknowledging that healthcare systems can influence the adoption of person-centred care. Page 29, lines 400-404.

6. Conclusions

Slightly vague; specific actionable recommendations could strengthen the conclusion.

Should explicitly state the limitations impacting interpretation.

We have made significant revisions to the conclusion to provide more actionable recommendations, taking into account the limitations of the review. See page 34-35, lines 531-546.

7. References

•Update and standardize all references to meet journal formatting guidelines.

•Ensure completeness with full bibliographic details.

•Include DOIs to improve accessibility.

•Maintain consistency in author listing, journal abbreviations, and sequential ordering.

Done

•Consider adding additional recent empirical studies directly related to leadership education outcomes to strengthen the literature base.

We have added the following references:

Parry K, Bryman A. Leadership in organizations. In: Clegg S, Hardy C, Lawrence T, Nord W, editors. The SAGE handbook of organization studies. London: SAGE Publ; 2013.

Guibert-Lacasa C, Vázquez-Calatayud M. Nurses’ clinical leadership in the hospital setting: A systematic review. Journal of Nursing Management. 2022;30:913-25.

Sparr J, Knipfer K, Willems F. How leaders can get the most out of formal training: The significance of feedback‐seeking and reflection as informal learning behaviors. Human Resource Development Quarterly. 2017;28(1):29-54.

Phillipson J, Pinto AC, Kingsley-Smith H, Krachler N, McGivern G, Lyons O. Leadership training in healthcare: a systematic umbrella review. BMJ Lead. 2025.

Reviewer #2: It was a pleasure reading this article. The article is overall well written and tells an interesting story of a relevant issue.

Thank you!

Main points:

I would welcome if the abstract would foreshadow the type of criteria used for inclusion.

We have added information on this in the abstract on page 2, lines 28-30.

The concepts around Person centred care are well described in the introduction. The concept of leadership education could however be stronger introduced to follow more logically towards the research question. You could map out the so what question of your research more in the end of the introduction.

We appreciate this insight, and we have added information on leadership on page 4, lines 74-77, and tried to map out the rationale for the review on pages 4-5, lines- 88-101.

Personally I would move the statement on the term 'stakeholders' to the methods.

We have now revised and moved this statement to the methods section under Data extraction process, pages 9-10, lines 188-190.

In the methods, I miss a more thorough description of the data analysis, e.g. how was it performed, who was involved, how were the presented categories decided upon.

We have elaborated on the analysis, providing information on how we performed the analysis (rather than synthesis), who was involved, and how the presented categories were decided upon to avoid interpretive synthesis. Please see page 10, lines 192-200.

The search strategy is sound and I applaud the inclusion of an information scientist. My only question is why only formal activities were included in the search. The lack of informal learning activities should be addressed in the discussion, or you should consider adding this to your search.

We appreciate your recognition of the involvement of an information scientist in developing the search strategy. While our research questions focused on education, which primarily highlights formal learning activities, we acknowledge the importance of informal learning in leadership development. A clarifying sentence has been added in the introduction (page 4, lines 90–93). We have also discussed how our definition of education may influence the searches on page 33, lines 515–517. The search strategy was carefully constructed to capture both formal education and informal learning activities relevant to person-centred care. For example, terms such as (MH learning+), (TI change OR AB change), and (TI implement* OR AB implement*) were included to reflect informal learning following leadership programmes.

The search strategy seems to be limited to English language which presents a misalignment with what you describe in the text.

Thank you for this comment which highlights a risk for misinterpretation in our reporting of the detailed search strategy. After discussing this with the information specialist involved in the search for literature, we have added information as part of Table 1 on pages 7-8, and we have added a table footnote on page 8, lines 149-150. As a clarification, we have also added the rationale for this decision on page 8, lines-161-162.

I very much appreciate the overview given in Table 2

Thank you!

Results are overall well described. I would however move the part on Knowledge gaps and future research to the Discussion or highlight more neatly which is part of your own analysis and conclusion and what has been suggested by the included articles. Currently the findings seems too intertwined with the areas for future research.

Thank you, we have revised the heading for this section of the results, to clarify that the knowledge gaps presented there are the ones presented in the included publications, see page 27, lin 360. We have also removed the last sentence: ”This highlights the need for more inclusive research that incorporates these perspectives.” and incorporated it in the discussion as that reflects our interpretation of the findings.

While the discussion addresses relevant points, i would suggest having another critical look at how you can structure it better so that the summary of findings, their reflecting in light of current research and educational practice aligns and what practical and scientific implications you see.

Thank you for this advice, we have revised the discussion with an introduction to the structure of the discussion (page 28, lines 384-387) and we have made revisions to clarify the findings in relation to current research and educational practice, see text highlighted in yellow in the results discussion on pages 28-32. The conclusion has been significantly revised to clarify the practical and scientific implications, pages 34-35, lines531-546.

Minor points:

There is a small typo in the abstract under Setting, where education should be listed in singular instead of plural.

We have corrected this.

Attachment Submitted filename: Response to reviewers.docx

# Peer review: Decision Letter 1

**DOI:** 10.1371/journal.pone.0350991.r003

**Authors:** Amal Diab Ghanem Atalla

15 Dec 2025

PONE-D-25-20590R1

Leadership education to support person-centred health and social care: a scoping review of empirical literature

PLOS One

Dear Dr. Lood,

Thank you for submitting your manuscript to PLOS ONE. After careful consideration, we feel that it has merit but does not fully meet PLOS ONE’s publication criteria as it currently stands. Therefore, we invite you to submit a revised version of the manuscript that addresses the points raised during the review process.

==============================

**ACADEMIC EDITOR:**

Dear Author,

Thank you for submitting your manuscript to our Journal. After careful evaluation by our reviewers and the editorial team, we have determined that your manuscript has merit but requires substantial revisions before it can be considered for publication. The reviewers have identified several key areas that need significant improvement, including [briefly mention the main concerns, e.g., methodological limitations, insufficient data analysis, unclear presentation of results, or inadequate discussion]. We encourage you to carefully address each of the reviewers' comments and provide a detailed response outlining the changes made. If any suggestions cannot be implemented, please provide a clear justification. Given the extent of the required revisions, your manuscript will undergo another round of peer review upon resubmission. Please ensure that your revised manuscript adheres to the journal’s formatting and reporting guidelines. We appreciate your efforts and look forward to receiving your revised manuscript. Please do not hesitate to reach out if you require any clarification.

Best regards,

[Amal Diab Ghanem Atalla]

[Academic editor]

==============================

Please submit your revised manuscript by Jan 26 2026 11:59PM. If you will need more time than this to complete your revisions, please reply to this message or contact the journal office at plosone@plos.org. When you're ready to submit your revision, log on to https://www.editorialmanager.com/pone/ and select the 'Submissions Needing Revision' folder to locate your manuscript file.

Please include the following items when submitting your revised manuscript:

- A letter that responds to each point raised by the academic editor and reviewer(s). You should upload this letter as a separate file labeled 'Response to Reviewers'.
- A marked-up copy of your manuscript that highlights changes made to the original version. You should upload this as a separate file labeled 'Revised Manuscript with Track Changes'.
- An unmarked version of your revised paper without tracked changes. You should upload this as a separate file labeled 'Manuscript'.

If you would like to make changes to your financial disclosure, please include your updated statement in your cover letter. Guidelines for resubmitting your figure files are available below the reviewer comments at the end of this letter.

If applicable, we recommend that you deposit your laboratory protocols in protocols.io to enhance the reproducibility of your results. Protocols.io assigns your protocol its own identifier (DOI) so that it can be cited independently in the future. For instructions see: https://journals.plos.org/plosone/s/submission-guidelines#loc-laboratory-protocols. Additionally, PLOS ONE offers an option for publishing peer-reviewed Lab Protocol articles, which describe protocols hosted on protocols.io. Read more information on sharing protocols at https://plos.org/protocols?utm_medium=editorial-email&utm_source=authorletters&utm_campaign=protocols.

We look forward to receiving your revised manuscript.

Kind regards,

Amal Diab Ghanem Atalla

Academic editor

PLOS One

Journal Requirements:

If the reviewer comments include a recommendation to cite specific previously published works, please review and evaluate these publications to determine whether they are relevant and should be cited. There is no requirement to cite these works unless the editor has indicated otherwise.

Additional Editor Comments:

Dear Author,

Thank you for submitting your manuscript to our Journal. After careful evaluation by our reviewers and the editorial team, we have determined that your manuscript has merit but requires substantial revisions before it can be considered for publication.

The reviewers have identified several key areas that need significant improvement, including [briefly mention the main concerns, e.g., methodological limitations, insufficient data analysis, unclear presentation of results, or inadequate discussion]. We encourage you to carefully address each of the reviewers' comments and provide a detailed response outlining the changes made. If any suggestions cannot be implemented, please provide a clear justification.

Given the extent of the required revisions, your manuscript will undergo another round of peer review upon resubmission. Please ensure that your revised manuscript adheres to the journal’s formatting and reporting guidelines.

We appreciate your efforts and look forward to receiving your revised manuscript. Please do not hesitate to reach out if you require any clarification.

Best regards,

[Amal Diab Ghanem Atalla]

[Academic editor]

[Note: HTML markup is below. Please do not edit.]

Reviewers' comments:

Reviewer's Responses to Questions

**Comments to the Author**

1. If the authors have adequately addressed your comments raised in a previous round of review and you feel that this manuscript is now acceptable for publication, you may indicate that here to bypass the “Comments to the Author” section, enter your conflict of interest statement in the “Confidential to Editor” section, and submit your "Accept" recommendation.

Reviewer #3: All comments have been addressed

**********

2. Is the manuscript technically sound, and do the data support the conclusions?

The manuscript must describe a technically sound piece of scientific research with data that supports the conclusions. Experiments must have been conducted rigorously, with appropriate controls, replication, and sample sizes. The conclusions must be drawn appropriately based on the data presented.

Reviewer #3: Yes

**********

3. Has the statistical analysis been performed appropriately and rigorously?

Reviewer #3: Yes

**********

4. Have the authors made all data underlying the findings in their manuscript fully available?

The PLOS Data policy requires authors to make all data underlying the findings described in their manuscript fully available without restriction, with rare exception (please refer to the Data Availability Statement in the manuscript PDF file). The data should be provided as part of the manuscript or its supporting information, or deposited to a public repository. For example, in addition to summary statistics, the data points behind means, medians and variance measures should be available. If there are restrictions on publicly sharing data—e.g. participant privacy or use of data from a third party—those must be specified.

Reviewer #3: (No Response)

**********

5. Is the manuscript presented in an intelligible fashion and written in standard English?

PLOS ONE does not copyedit accepted manuscripts, so the language in submitted articles must be clear, correct, and unambiguous. Any typographical or grammatical errors should be corrected at revision, so please note any specific errors here.

Reviewer #3: Yes

**********

6. Review Comments to the Author

Please use the space provided to explain your answers to the questions above. You may also include additional comments for the author, including concerns about dual publication, research ethics, or publication ethics. (Please upload your review as an attachment if it exceeds 20,000 characters)

Reviewer #3: Title and Keywords

Title length: The full title is overly long and could be more concise while retaining clarity.

Keywords: Some keywords (e.g., “manager”) are too generic and do not reflect the scope of the review; missing terms like “leadership training” or “scoping review.”

Abstract

Lack of clarity on study type: The abstract does not clearly specify the types of empirical studies included (qualitative, quantitative, mixed methods).

Insufficient mention of limitations: While limitations are mentioned, they are vague and not linked to interpretation of findings.

Overly descriptive: The abstract reads more like a summary than a structured scientific abstract; lacks concise statements on methodology and implications.

Typographical error: “educations” should be singular (“education”) under Setting.

Introduction

Insufficient theoretical framing: Leadership education is not introduced strongly enough; the link between person-centred care and leadership education lacks logical flow.

Missing research questions upfront: Research questions appear late; hypotheses are absent (though scoping reviews typically do not test hypotheses, this should be clarified earlier).

Contextual gap: Limited discussion of global healthcare system differences affecting generalizability.

Methods

Search strategy reporting: Although Table 1 includes search strings, the rationale for excluding “patient-centred care” MeSH term is weak and could bias results.

Language bias: Restriction to certain languages introduces bias; justification is minimal.

Data analysis description: The process for coding and category development is underexplained; lacks detail on inter-rater reliability or validation.

Interest holder definition: The term “interest holder” is introduced late and should be clarified earlier in Methods.

Results

Insufficient detail on interventions: Descriptions of educational interventions are brief and lack depth (e.g., pedagogical strategies, theoretical underpinnings).

Heterogeneity not addressed: Wide variation in study designs and contexts is noted but not analyzed for implications.

Tables: Table 3 is dense and lacks clarity; inconsistent formatting of education length and participant details.

Discussion

Weak critical analysis: Discussion does not sufficiently critique methodological limitations (e.g., small sample sizes, lack of control groups).

Contextual factors missing: No exploration of how healthcare system differences influence applicability.

Integration with existing literature: Limited synthesis with broader leadership education research; theoretical integration is weak.

Conclusions

Vague recommendations: Conclusions are broad and lack actionable steps for educators or policymakers.

Limitations not linked to interpretation: While limitations are mentioned, their impact on findings is not clearly articulated.

References

Formatting inconsistencies: Some references lack proper DOI formatting; inconsistent use of italics and punctuation.

Currency of literature: Although recent references were added, some seminal works on leadership education are missing.

Redundancy: Multiple references from the same authors without clear justification for inclusion.

9. Figures and Tables

Figure 1 (PRISMA): Poor resolution and unclear labeling; does not meet journal quality standards.

Tables: Overly complex, lack clear headings, and inconsistent terminology (e.g., “education participants” vs. “study participants”).

Language and Style

Typographical errors: Examples include “Northen Europe” instead of “Northern Europe.”

Overuse of passive voice: Reduces clarity and engagement.

Repetitive phrasing: Frequent repetition of “person-centred care” without variation; could be condensed for readability.

Journal style compliance: Some sections (e.g., headings, abbreviations) do not fully align with PLOS ONE formatting guidelines.

**********

7. PLOS authors have the option to publish the peer review history of their article (what does this mean?). If published, this will include your full peer review and any attached files.

If you choose “no”, your identity will remain anonymous but your review may still be made public.

**Do you want your identity to be public for this peer review?** For information about this choice, including consent withdrawal, please see our Privacy Policy.

Reviewer #3: **Yes:** Associate Professor/ Marwa Samir Sorour

**********

[NOTE: If reviewer comments were submitted as an attachment file, they will be attached to this email and accessible via the submission site. Please log into your account, locate the manuscript record, and check for the action link "View Attachments". If this link does not appear, there are no attachment files.]

To ensure your figures meet our technical requirements, please review our figure guidelines: https://journals.plos.org/plosone/s/figures

You may also use PLOS’s free figure tool, NAAS, to help you prepare publication quality figures: https://journals.plos.org/plosone/s/figures#loc-tools-for-figure-preparation.

NAAS will assess whether your figures meet our technical requirements by comparing each figure against our figure specifications.

# Author comment: Author response to Decision Letter 2

**DOI:** 10.1371/journal.pone.0350991.r004

4 Feb 2026

The authors would like to express their gratitude for the reviewer’s work and thoughtful comments. We have revised the manuscript extensively in response to all comments, and we believe that these revisions have substantially strengthened the clarity, coherence, and scientific rigour of the paper. Below, we respond to each comment in turn. Major changes made to the manuscript are highlighted in yellow.

Reviewer #3: Title and Keywords

Title length: The full title is overly long and could be more concise while retaining clarity.

The title has been retained as originally submitted, as it corresponds to the title registered in the review protocol and is consistent with recommended reporting guidelines. The title also falls well below the maximum length permitted by the journal. For these reasons, we have chosen not to modify the title.

Keywords: Some keywords (e.g., “manager”) are too generic and do not reflect the scope of the review; missing terms like “leadership training” or “scoping review.”

We have revised the keywords accordingly. The term “manager” has been removed and “leadership training” has been added. We did not add “scoping review” as a keyword, as that is clearly stated in the title.

Abstract

Lack of clarity on study type: The abstract does not clearly specify the types of empirical studies included (qualitative, quantitative, mixed methods).

The abstract has been revised to explicitly state that qualitative, quantitative, and mixed-methods empirical studies were eligible for inclusion, see page 2, lines 33-34.

Insufficient mention of limitations: While limitations are mentioned, they are vague and not linked to interpretation of findings.

We have revised the abstract to provide a clearer and more explicit description of key limitations and to link these directly to the interpretation of the findings. See page 2, lines 42-47.

Overly descriptive: The abstract reads more like a summary than a structured scientific abstract; lacks concise statements on methodology and implications.

The abstract has been substantially revised to follow a more scientific format, we hope you find it more structured and concise. See pages 2-23.

Typographical error: “educations” should be singular (“education”) under Setting.

Thank you for noting this. The terminology has been corrected throughout the manuscript to education or education interventions, as appropriate.

Introduction

Insufficient theoretical framing: Leadership education is not introduced strongly enough; the link between person-centred care and leadership education lacks logical flow.

The introduction has been revised to strengthen the theoretical framing of leadership and to improve the logical flow between person-centred care, leadership, and leadership education. See pages 3-5, lines 61-64, 70-72, 77-79, 82-88, 90-102, and 111-119.

Missing research questions upfront: Research questions appear late; hypotheses are absent (though scoping reviews typically do not test hypotheses, this should be clarified earlier).

We have clarified the rationale for a scoping review approach on page 6, lines 126-128, for which hypothesis testing is not appropriate. The research questions are presented following the stated aim, in accordance with scoping review methodology.

Contextual gap: Limited discussion of global healthcare system differences affecting generalizability.

We have expanded the introduction to include healthcare system variation and its implications for leadership education. See, pages 5-6, lines 121-125.

Methods

Search strategy reporting: Although Table 1 includes search strings, the rationale for excluding “patient-centred care” MeSH term is weak and could bias results.

We have strengthened the justification for excluding this term in the Methods section (page 8, lines186-191) and further elaborated on the conceptual distinction between patient-centred and person-centred care in the introduction (page 3-4, lines 66-79) and we discuss it on pages 33-34, lines 574-578

Language bias: Restriction to certain languages introduces bias; justification is minimal.

The rationale for language restrictions has been clarified in the methods (page 9, lines 175-177), and the implications of potential language bias are now explicitly addressed in the discussion, see page 34, lines 578-581.

Data analysis description: The process for coding and category development is underexplained; lacks detail on inter-rater reliability or validation.

The Data analysis section has been revised to provide a clearer description of the process, including iterative category development, and validation procedures, see page 11. We acknowledge the reviewer’s comment regarding inter-rater reliability. In line with JBI guidance and qualitative methodology, we ensured rigour through triangulation and consensus-based category development, which we have now clarified in the Data analysis on page 11.

Interest holder definition: The term “interest holder” is introduced late and should be clarified earlier in Methods.

The definition of interest holder is clarified in the methods section, and its use is consistent throughout the manuscript.

Results

Insufficient detail on interventions: Descriptions of educational interventions are brief and lack depth (e.g., pedagogical strategies, theoretical underpinnings).

The results section has been substantially revised and restructured to provide more detailed and analytically organised descriptions of key content, educational methods, reported results, and knowledge gaps.

Heterogeneity not addressed: Wide variation in study designs and contexts is noted but not analyzed for implications.

We now explicitly address heterogeneity in study design, context, and intervention characteristics and discuss its implications for interpretation and transferability in the Discussion section on pages 28-31, lines 431-447, 513-525, and in the Strengths and limitations on page 33, lines 552-559 and 563-566.

Tables: Table 3 is dense and lacks clarity; inconsistent formatting of education length and participant details.

Table 3 has been revised for clarity and consistency. A streamlined version is now included in the main manuscript, while more details have been added to the text describing the results. In line with this comment, we have also revised table 2, omitting details on study participants as this may confuse the readers. our focus is on education participants (i.e., leaders), whereas study participants sometimes included direct care staff and patients.

Discussion

Weak critical analysis: Discussion does not sufficiently critique methodological limitations (e.g., small sample sizes, lack of control groups).

We have strengthened the critical analysis of methodological limitations, including small sample sizes, lack of control groups, reliance on self-reported outcomes, and implications for interpretation (see revised discussion and strengths and limitations sections). See Strengths and limitations on pages 32-34.

Contextual factors missing: No exploration of how healthcare system differences influence applicability.

We have expanded both the introduction and discussion to include healthcare system differences and their influence on applicability and generalisability. See for example pages 5-6, lines 121-125, and page 28, lines 435-447.

Integration with existing literature: Limited synthesis with broader leadership education research; theoretical integration is weak.

The discussion has been revised to more explicitly integrate findings with broader leadership education and leadership theory literature, including recent work on leadership plurality, overlap between leadership approaches, context dependency, and co-leadership in integrated care settings. For example pages 29-30, lines 471-484 and the reference lists for updated references.

Conclusions

Vague recommendations: Conclusions are broad and lack actionable steps for educators or policymakers.

The conclusions section has been substantially revised to provide clearer and more actionable implications for educators, researchers, and policymakers. See pages 34-35.

Limitations not linked to interpretation: While limitations are mentioned, their impact on findings is not clearly articulated.

We now explicitly link key limitations to interpretation of findings both in the Strengths and Limitations and Conclusions sections.

References

Formatting inconsistencies: Some references lack proper DOI formatting; inconsistent use of italics and punctuation.

All references have been carefully reviewed to ensure full compliance with formatting requirements.

Currency of literature: Although recent references were added, some seminal works on leadership education are missing.

Additional recent and relevant literature on leadership education and leadership theory has been incorporated to strengthen the theoretical integration of the manuscript, informed by scholarly consultation acknowledged in the paper.

Redundancy: Multiple references from the same authors without clear justification for inclusion.

We have reviewed the reference list to ensure that multiple citations from the same authors are justified by distinct contributions.

9. Figures and Tables

Figure 1 (PRISMA): Poor resolution and unclear labeling; does not meet journal quality standards.

Figure 1 has been revised to improve resolution and labelling in accordance with journal standards.

Tables: Overly complex, lack clear headings, and inconsistent terminology (e.g., “education participants” vs. “study participants”).

The tables have been revised to improve clarity, simplify structure, and ensure consistent terminology throughout (e.g., standardising the use of education participants and excluding information on study participants in table 2, as explained in our previous reply). Headings have been clarified and table content streamlined to enhance readability and alignment with the manuscript text.

Language and Style

Typographical errors: Examples include “Northen Europe” instead of “Northern Europe.”

We hope that all typographical errors have been corrected.

Overuse of passive voice: Reduces clarity and engagement.

The manuscript has undergone careful language revision to reduce unnecessary passive constructions and repetitive phrasing, improving readability and flow.

Repetitive phrasing: Frequent repetition of “person-centred care” without variation; could be condensed for readability.

We acknowledge the reviewer’s comment. Given that person-centred care is the core concept of the manuscript and central to its theoretical and empirical focus, we have retained consistent terminology to ensure conceptual clarity and avoid ambiguity. Where possible, minor wording adjustments have been made to improve flow without substituting the term in ways that could alter its meaning.

Journal style compliance: Some sections (e.g., headings, abbreviations) do not fully align with PLOS ONE formatting guidelines.

All sections, headings, abbreviations, and formatting have been reviewed and adjusted to align with submission guidelines.

We thank the reviewer again for the constructive feedback, which has significantly improved the quality and clarity of the manuscript.

Attachment Submitted filename: Rebuttal letter R2.docx

# Editor report: Decision Letter 2

**DOI:** 10.1371/journal.pone.0350991.r005

**Authors:** Ahmad Al-Nawafleh

22 May 2026

Leadership education to support person-centred health and social care: a scoping review of empirical literature

PONE-D-25-20590R2

Dear Dr. Lood,

We’re pleased to inform you that your manuscript has been judged scientifically suitable for publication and will be formally accepted for publication once it meets all outstanding technical requirements.

Within one week, you’ll receive an e-mail detailing the required amendments. When these have been addressed, you’ll receive a formal acceptance letter and your manuscript will be scheduled for publication.

An invoice will be generated when your article is formally accepted. Please note, if your institution has a publishing partnership with PLOS and your article meets the relevant criteria, all or part of your publication costs will be covered. Please make sure your user information is up-to-date by logging into Editorial Manager at Editorial Manager® and clicking the ‘Update My Information' link at the top of the page. For questions related to billing, please contact billing support.

If your institution or institutions have a press office, please notify them about your upcoming paper to help maximize its impact. If they’ll be preparing press materials, please inform our press team as soon as possible -- no later than 48 hours after receiving the formal acceptance. Your manuscript will remain under strict press embargo until 2 pm Eastern Time on the date of publication. For more information, please contact onepress@plos.org.

Kind regards,

Ahmad H. Al-Nawafleh, Ph.D, MPA, CI, RN

Academic Editor

PLOS One

Additional Editor Comments (optional):

Reviewers' comments:

<!--a=1

# Editor report: Acceptance letter

**DOI:** 10.1371/journal.pone.0350991.r006

**Authors:** Ahmad Al-Nawafleh

PONE-D-25-20590R2

PLOS One

Dear Dr. Lood,

I'm pleased to inform you that your manuscript has been deemed suitable for publication in PLOS One. Congratulations! Your manuscript is now being handed over to our production team.

At this stage, our production department will prepare your paper for publication. This includes ensuring the following:

* All references, tables, and figures are properly cited

* All relevant supporting information is included in the manuscript submission,

* There are no issues that prevent the paper from being properly typeset

You will receive further instructions from the production team, including instructions on how to review your proof when it is ready. Please keep in mind that we are working through a large volume of accepted articles, so please give us a few days to review your paper and let you know the next and final steps.

Lastly, if your institution or institutions have a press office, please let them know about your upcoming paper now to help maximize its impact. If they'll be preparing press materials, please inform our press team within the next 48 hours. Your manuscript will remain under strict press embargo until 2 pm Eastern Time on the date of publication. For more information, please contact onepress@plos.org.

You will receive an invoice from PLOS for your publication fee after your manuscript has reached the completed accept phase. If you receive an email requesting payment before acceptance or for any other service, this may be a phishing scheme. Learn how to identify phishing emails and protect your accounts at https://explore.plos.org/phishing.

If we can help with anything else, please email us at customercare@plos.org.

Thank you for submitting your work to PLOS ONE and supporting open access.

Kind regards,

PLOS ONE Editorial Office Staff

on behalf of

Prof. Ahmad H. Al-Nawafleh

Academic Editor

PLOS One
